# PHR‐Mediated Pi Starvation Response Mobile Messenger RNAs Represent Noncoding Transcripts in Recipient Tissues

**DOI:** 10.1002/advs.202502911

**Published:** 2025-11-30

**Authors:** Weiguo Dong, Shuman Wang, Chao Liu, Lulu Qiao, Kai Liu, Qiongli Jin, Xiaorong Mo, Xingxing Shen, Keke Yi, Zhiye Wang

**Affiliations:** ^1^ State Key Laboratory of Plant Environmental Resilience, College of Life Sciences Zhejiang University Hangzhou Zhejiang 310058 China; ^2^ Key Laboratory of Biology of Crop Pathogens and Insects of Zhejiang Province, College of Agriculture and Biotechnology Zhejiang University Hangzhou Zhejiang 310058 China; ^3^ State Key Laboratory of Efficient Utilization of Arid and Semi‐arid Arable Land in Northern China/Key Laboratory of Plant Nutrition and Fertilizer, Ministry of Agriculture, Institute of Agricultural Resources and Regional Planning Chinese Academy of Agricultural Sciences Beijing 100097 China

**Keywords:** mRNA long‐distance transport, noncoding transcripts, phosphate starvation response, PHR, RNA structure

## Abstract

Nutritional deficiency for phosphate (Pi) or nitrogen triggers long‐distance transport of specific mobile mRNAs, but little is known about the underlying regulation, function, and structural features of these mobile nutrient starvation‐specific mRNAs. Here, heterografting and high‐throughput sequencing are used to explore the landscape of long‐distance mRNA transport in Arabidopsis under normal and Pi‐starvation conditions. Hundreds of Pi starvation response (PSR)–specific mobile mRNAs are discovered, although their abundance is constant between normal and Pi starvation conditions. The mobility of these PSR‐specific mobile mRNAs is largely dependent on PHOSPHATE STARVATION RESPONSE (PHR) function, based on mutant analysis. Notably, translatome analysis and transgenic experiments reveal that these PHR‐mediated PSR‐specific mobile mRNAs are translated in donor tissues, but not in recipient tissues. Further, these mobile mRNAs harbored a more unfolded 5′ UTR than nonmobile mRNAs, non‐PSR‐mobile mRNAs, and nitrogen starvation–response mobile mRNAs. The study reveals that these PSR‐specific mobile mRNAs are transported to recipient tissues to carry out a function independently of their encoded protein.

## Graphical abstract

Mobile messenger RNAs (mRNAs) can travel long distances, serving as systemic signals that participate in plant growth and stress acclimation. This work determines that PHR proteins mediate the Pi starvation response (PSR)‐specific long‐distance transport of mRNAs and that these PSR‐specific mobile mRNAs represent noncoding transcripts in recipient tissues.

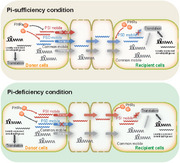

## 1 Introduction

Plants acclimate to their environment by producing long‐distance signals for intercellular and intra‐organismal communication to coordinate development and environmental responses.^[^ ^1^ ^]^ These long‐distance signal molecules include calcium, metabolites, phytohormones, peptides, and small RNAs.^[^ ^2^, ^3^ ^]^ Emerging evidence showed that numerous mRNA molecules are transported over long distances within the plant to target specific tissues, where they function as non‐cell autonomous informational macromolecules.^[^ ^4^, ^5^ ^]^ Thousands of mRNAs move between tissues^[^ ^6^, ^7^, ^8^ ^]^ or between hosts and parasitic plants,^[^ ^9^ ^]^ even crossing kingdoms between hosts and microbes.^[^ ^10^ ^]^ Of note, nutrient limitation, such as inorganic phosphate (Pi) or nitrogen deficiency, triggers long‐distance transport of specific mRNAs.^[^ ^7^, ^8^ ^]^

Although many mobile mRNAs have been identified, the functions of a few such long‐distance mobile mRNAs in signaling have been reported. These long‐distance transport‐associated functions include leaf development,^[^ ^11^, ^12^ ^]^ root architecture development,^[^ ^13^, ^14^ ^]^ pollen tube production,^[^ ^15^, ^16^ ^]^ flowering time,^[^ ^17^ ^]^ and defense responses.^[^ ^10^ ^]^ Moreover, several studies have demonstrated the translation of mobile mRNAs in recipient tissues or cells using reporter constructs, mass spectrometry, or translating ribosome affinity purification (TRAP) analyses.^[^ ^8^, ^9^, ^12^, ^14^, ^18^ ^]^ To date, a translatome‐based validation of mobile mRNA translation in plants has not yet been performed. In addition, RNA sequence motifs and RNA modifications have been reported as specific codes for long‐distance mRNA transport.^[^ ^12^, ^14^, ^19^ ^]^ Some mobile mRNAs present a transfer RNA (tRNA)‐like structure (TLS) required for their mobility, and dicistronic mRNA:tRNA transcripts are enriched among graft‐mobile mRNAs (≈12%).^[^ ^12^ ^]^ In recent work, the nuclease gene *Cas9* and its associated single guide RNAs (sgRNAs) were shown to be mobilized by fusing these sequences to TLS motifs.^[^ ^20^ ^]^ Other studies showed that the mobility of *GIBBERELLIC ACID INSENSITIVE* (*GAI*) transcripts in Arabidopsis (*Arabidopsis thaliana*) requires a specific RNA motif in the 3′ sequence of the RNA.^[^ ^21^, ^22^ ^]^ In addition, the epitranscriptomic mark 5‐methylcytosine (m^5^C) influences mRNA transport.^[^ ^14^, ^19^ ^]^ Overall, it remains unclear how specific mRNAs are transported over long distances or how their transport relates to their biological functions, particularly for acclimation to stress.

Phosphorus (P) is an essential macronutrient for plant growth and development, as it is a constituent of key macromolecules such as nucleic acids, phospholipids, and numerous intermediary metabolites, and is important in signaling via kinases and phosphatases. Although plants take up P from the rhizosphere in the form of Pi, it is often fixed in the soil and is not bioavailable.^[^ ^23^, ^24^ ^]^ To cope with Pi limitation, plants have evolved numerous adaptive developmental and metabolic responses.^[^ ^25^, ^26^ ^]^ The Pi starvation response (PSR) entails interorgan communication of Pi levels via systemic signaling.^[^ ^27^ ^]^ In *Arabidopsis*, microRNA399 (miR399) acts as a long‐distance signal of Pi starvation.^[^ ^28^, ^29^ ^]^ Pi starvation–induced (PSI) mature miR399 is transported from shoots to roots and cleaves *PHOSPHATE2* (*PHO2*) mRNA, encoding a ubiquitin‐conjugating E2 enzyme.^[^ ^30^ ^]^ The depletion of *PHO2* transcripts causes the accumulation of the Pi transporters PHOSPHATE TRANSPORTER1 (PHT1) and PHO1, greater Pi acquisition, and root‐to‐shoot translocation of Pi.^[^ ^31^, ^32^, ^33^ ^]^ In addition to miRNA movement, Pi starvation also induces the movement of specific mRNAs,^[^ ^7^, ^8^ ^]^ although their biological functions and RNA structural features are unknown.

PHOSPHATE STARVATION RESPONSE (PHR) proteins are MYB coiled‐coil transcription factors (TFs) acting as key positive regulators of the PSR.^[^ ^34^, ^35^ ^]^ PHR1 and PHR1‐LIKE (PHL) TFs regulate PSI transcriptional reprogramming,^[^ ^36^ ^]^ chromatin accessibility,^[^ ^37^ ^]^ lipid remodeling,^[^ ^38^ ^]^ metabolism,^[^ ^39^ ^]^ and mycorrhizal symbiosis.^[^ ^40^ ^]^ PHRs also regulate the induction of *MIR399* expression under Pi limitation.^[^ ^41^, ^42^ ^]^ However, it is unknown whether PHRs regulate PSR‐induced long‐distance transport of mRNAs.

Here, using heterografting and bioinformatics analysis, we identified hundreds of mobile mRNAs in response to Pi starvation. The mobility, but not the abundance, of these PSR‐specific mobile transcripts was strongly dependent on PHR function. TRAP‐seq analysis revealed that these PHR‐mediated PSR‐specific mobile mRNAs were translated in donor tissues, but not in recipient tissues, suggesting a protein‐independent function for these mobile RNAs in recipient tissues. We further developed transgenic plants and confirmed the Pi starvation–induced long‐distance mRNA transport and lack of translation of selected PHR‐mediated PSR‐specific mobile mRNAs in recipient tissues. RNA structural analysis showed that these PSR‐specific mobile transcripts harbored a more unfolded 5′ untranslated region (UTR) than that of nonmobile transcripts, non‐PSR‐mobile transcripts, or nitrogen starvation–responsive transcripts. These results reveal a previously unknown function for PHRs in regulating long‐distance mRNA transport in response to Pi starvation and suggest that PSR‐specific mRNAs might play a noncoding function in recipient tissues.

## 2 Results

### 2.1 Transcriptome‐Wide Profiling of Mobile Transcripts under Pi‐Sufficient and Pi‐Deficient Conditions

**Figure 1:**
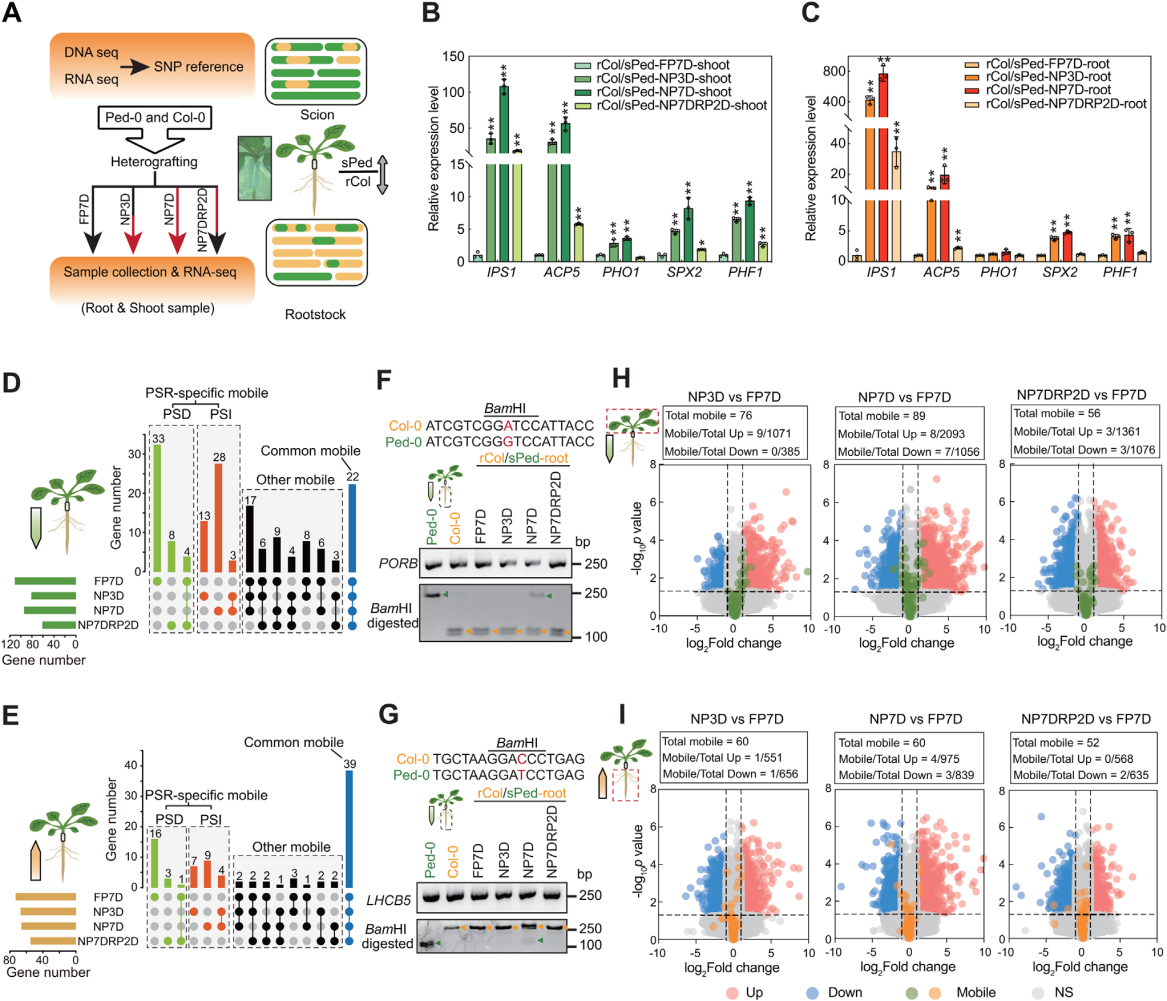
Phosphate starvation induces long‐distance RNA transport. A) Outline of the experimental strategy and diagram of the reciprocal hypocotyl grafting. Chimeric seedlings were generated by hypocotyl grafting of the highly polymorphic *Arabidopsis* accessions Col‐0 (Col) and Ped‐0 (Ped) before being subjected to different regimes of inorganic phosphate (Pi) deficiency. rCol/sPed, heterografting between Ped‐0 shoot (green) and Col‐0 root (orange). Green and orange boxes indicate the locally expressed transcripts in the scion and rootstock, respectively. Green boxes marked with yellow in the scion indicate the mobile transcript from the rootstock and vice versa. B,C) Expression levels of PSR genes from RNA‐seq in response to the Pi deficiency treatments in (B) shoot and C) root samples of rCol/sPed. The values are means ± standard deviation (SD) from three biological replicates. *, *P* < 0.05, **, *P* < 0.01; two‐tailed Student's *t*‐test. D,E) Upset diagrams showing the number of identified mobile transcripts moving from D) shoots to roots or E) roots to shoots as a function of Pi deficiency conditions. Numbers of Pi starvation–induced (PSI) and Pi starvation–decreased (PSD) PSR‐specific mobile RNAs are shown in red and green, respectively. Numbers of common mobile RNAs among normal and −Pi conditions are shown in blue. Numbers of the other mobile RNAs are shown in black. F,G) Confirmation of PSR‐specific mobile RNAs using RT‐PCR and CAPS markers specific for F) *PORB* or G) *LHCB5*. Green and orange arrows indicate the Ped‐0 and Col‐0 PCR products, respectively, after digestion with *Bam*HI. H,I) Volcano plots showing that the abundance of most mobile RNAs does not change under Pi deficiency treatments, for H) shoot‐to‐root and I) root‐to‐shoot mobile RNAs. Compared to the expression in FP7D, genes with at least 2‐fold higher or lower expression and *P*‐values <0.05 are indicated by red or blue circles, respectively. Gray circles indicate genes showing no difference. Green and orange circles indicate the mobile transcripts under different Pi deficiency treatments. FP7D, 7 days Pi‐sufficient; NP3D, 3 days Pi‐deficient; NP7D, 7 days Pi‐deficient; NP7DRP2D, 7 days Pi‐deficient, followed by 2 days Pi resupply conditions. r, root. s, shoot. Up, significantly upregulated. Down, significantly downregulated. NS, no significant difference.

To identify mobile long transcripts under normal and/or Pi‐starvation conditions, we adopted a method based on heterografts between two polymorphic *Arabidopsis* accessions (Col‐0 and Ped‐0) followed by high‐throughput sequencing and bioinformatics analysis^[^ ^8^ ^]^ to identify mobile RNAs based on accession‐specific single nucleotide polymorphisms (SNPs) (**Figure** **1**A). As this method requires high‐quality genome sequences, we re‐sequenced the Ped‐0 genome using long‐read (PacBio) and short‐read (Illumina) DNA sequencing to improve the accuracy of mobile RNA identification. We assembled the Ped‐0 genome *de novo*, consisting of 211 scaffolds with a high N50 value (18 521 859 bp) (Figure S1A, Supporting Information; see Experimental Section). In total, we obtained five main genomic scaffolds, the sizes of which were similar to the five chromosomes of Col‐0 (TAIR10) (Figure S1A, Supporting Information). Benchmarking Universal Single‐Copy Orthologs (BUSCOs)^[^ ^43^ ^]^ analysis revealed that 4,472 (97.3%) of the 4,596 Embryophyta single‐copy orthologous genes were captured in our Ped‐0 genome (Figure S1B, Supporting Information). We also used Illumina‐based transcriptome deep sequencing (RNA‐seq) to profile the expression landscape in Ped‐0 and Col‐0 under Pi‐sufficient or ‐deficient conditions, from which we predicted Ped‐0 transcripts. We thus annotated 27,639 genes in the new Ped‐0 genome, 26,072 (94.3%) of which were homologous to TAIR10 genes (Figure S1C, Supporting Information).

We used GATK HaplotypeCaller^[^ ^44^ ^]^ to identify SNPs between the Col‐0 and Ped‐0 genomes (Figure S1D, Supporting Information), following stringent selection criteria for confidently identifying accession‐specific SNPs. First, the SNP should pass our hard‐filtering criteria (see Experimental Section); second, it should be supported by the RNA‐seq and genomic data; third, it must have genotype‐specific alleles across the datasets; and fourth, the SNP site should be covered by at least 10 reads in both the RNA‐seq and the genomic sequence data. We excluded SNPs mapping to the mitochondria and chloroplast genomes to avoid false positives due to potential RNA editing events. The resulting SNPs satisfying these criteria were considered high‐confidence SNPs and were used as SNP reference sites for investigation. In total, we identified 394 009 high‐confidence SNP sites, which could unambiguously distinguish 25 810 transcripts (≈76.8% of the 33 602 known *Arabidopsis* genes) between Ped‐0 and Col‐0 (Figure S1E, Supporting Information). These distinguishable transcripts correspond to genes mapping evenly across the five chromosomes, indicating that our pipeline was unbiased (Figure S1F, Supporting Information).

We heterografted Ped‐0 and Col‐0 seedlings grown under Pi‐sufficient conditions at 7 days after germination (Col‐root/Ped‐shoot, hereafter as rCol/sPed) grown in Pi‐sufficient conditions and returned the grafts to Pi‐sufficient conditions for another 11 days. We then subjected these heterografts to various treatments: 7 days of Pi sufficiency (full Pi for 7 days [FP7D], serving as the control), 3 days of Pi deficiency (no Pi for 3 days [NP3D]), 7 days of Pi deficiency (NP7D), and 7 days of Pi deficiency followed by 2 days of Pi resupply (NP7DRP2D) (Figure 1A). In parallel, we conducted homografting of Ped‐0 (Ped‐root/Ped‐shoot [rPed/sPed]) and Col‐0 (Col‐root/Col‐shoot [rCol/sCol]) seedlings as a grafting control to rule out possible effects due to the physical manipulation. We harvested the roots and shoots from all seedlings at the end of the treatments as three biological replicates (>15 seedlings per biological replicate) per treatment. We confirmed the genotype of each collected tissue using a cleaved amplified polymorphic sequence (CAPS) marker distinguishing between Col‐0 and Ped‐0^[^ ^8^ ^]^ (Figure S2A, Supporting Information). We used these samples for RNA extraction and RNA‐seq.

We obtained 31–83 million clean reads for each biological replicate, of which >74.4% were uniquely mapped to the corresponding Col‐0 (TAIR10) or our new Ped‐0 genome (Table S1, Supporting Information). Clustering and principal component analysis (PCA) showed high reproducibility among the three biological replicates (Figure S2B,C, Supporting Information). To assess the effectiveness of the treatments, we examined the transcript levels of Pi starvation–response (PSR) marker genes. The expression of PSR marker genes from RNA‐seq showed the expected induction under different Pi deficiency conditions, in samples collected from homografts and heterografts (Figure 1B,C; Figure S2D and Tables S2 and S3, Supporting Information). These results also indicated comparable PSR signaling between Ped‐0 and Col‐0.

We next identified mobile transcripts using our custom pipeline from all grafted samples (i.e., rCol/sPed samples under all conditions) between Col‐0 and Ped‐0 (Figure S1D, Supporting Information). To accurately identify the accession‐specific SNP sites, we used Col‐0 and Ped‐0 homografted plants and Baymobil^[^ ^45^, ^46^ ^]^ software to exclude sequencing errors that may be mistaken as SNPs. The SNPs that fulfilled the criterion of log_10_(Bayesian factor) > 1 were considered high‐confidence SNPs (Figure S1D, Supporting Information). We then extracted all reads covering high‐confidence SNPs and mapped them to the corresponding reference to identify mobile transcripts. The transcripts detected in at least two of the three biological replicates were considered high‐confidence mobile transcripts, as done by previous studies^[^ ^7^ ^]^ (Figures S1D and S2E and Tables S4 and S5, Supporting Information), and were used for analysis. From our RNA‐seq analysis of heterografted samples from +Pi (FP7D and NP7DRP2D) and −Pi (NP3D and NP7D) conditions, we identified 241 mobile transcripts produced by 241 genes (Figure S2E and Table S5, Supporting Information). Of these, 147 were specifically transported from shoots to roots, 77 were specifically transported from roots to shoots, and 17 were transported bidirectionally (Figure S2E, Supporting Information). A comparison of our mobile RNAs to those of a previous study in *Arabidopsis* ^[^ ^8^ ^]^ revealed 56 common mobile transcripts (*P* = 1.23e−18, hypergeometric test, indicating significant overlap between these two datasets), despite differences in growth conditions (plates or hydroponic system with different nutrient regimes) and the stringency of variant calling (Figure S2F, Supporting Information). Notably, most (92.7% shoot‐to‐root and 86.2% root‐to‐shoot) of the mobile RNAs identified here were protein‐coding mRNAs, regardless of the direction of movement and treatment (Figure S2G and Table S5, Supporting Information). A similar scenario was noted in previous studies in *Arabidopsis*,^[^ ^8^ ^]^ cucumber (*Cucumis sativus*),^[^ ^7^ ^]^ and pumpkin (*Cucurbita maxima*).^[^ ^47^ ^]^ Altogether, we identified many long‐distance mobile transcripts between tissues, supporting the idea that long‐distance RNA transport is a widespread phenomenon among species.

### 2.2 Pi Deficiency Induces Long‐Distance Transport of Specific RNAs

We looked for long‐distance transport of RNAs specifically in response to Pi deficiency. We observed that Pi starvation alters long‐distance RNA mobility. Many RNAs were transported under both normal (+P) and Pi deficiency (−P) conditions (Figure 1D,E). However, among the shoot‐to‐root mobile RNAs, 45 and 44 RNAs specifically moved under +Pi (e.g., FP7D and NP7DRP2D) or −Pi (e.g., NP3D and NP7D) conditions, respectively (Figure 1D). Similarly, among the root‐to‐shoot mobile RNAs, 20 and 20 RNAs specifically moved under +P or −P conditions, respectively (Figure 1E). Furthermore, the number of PSR‐mobile transcripts under long‐term Pi starvation (NP7D) was greater than that under short‐term Pi starvation conditions (NP3D). The 2‐day Pi‐refeeding (NP7DRP2D) treatment did not fully rescue the abundance of mobile transcripts to that seen under the normal growth condition (FP7D) (Figure 1D,E). We randomly chose two of these PSR‐specific mobile RNAs with high (*PROTOCHLOROPHYLLIDE OXIDOREDUCTASE B* [*PORB*]) or sub‐average (*LIGHT HARVESTING COMPLEX OF PHOTOSYSTEM II 5* [*LHCB5*]) mobile abundance and assessed their long‐distance transport using CAPS marker analysis following RT‐PCR from total RNA extracted from each grafted sample. Indeed, the CAPS analysis confirmed the Pi deficiency–mediated long‐distance transport of these two mobile mRNAs (Figure 1F,G). These results show that Pi starvation induces dynamic changes in the abundance of mobile transcripts, and these changes intensify with longer duration of Pi deficiency stress.

In addition to the PSR‐specific mobile RNAs, ≈13.4% (22/164) of shoot‐to‐root and 41.5% (39/94) of root‐to‐shoot mobile RNAs were transported under both +P and −P conditions (Figure 1D,E). We wondered whether the transport efficiency of those common mobile RNAs was also influenced by Pi deficiency. To test this idea, we calculated a mobile index (MI) as the ratio between the abundance of mobile RNAs in the recipient tissue and their corresponding RNA abundance in the donor tissue to assess the transport efficiency of a mobile transcript. The MI of 19 (accounting for 86.4% of shoot‐to‐root) and 24 (accounting for 61.5% of root‐to‐shoot) common mobile RNAs was altered under Pi deficiency (Figure S3 and Table S6, Supporting Information). Specifically, the MIs of 15 shoot‐to‐root and 16 root‐to‐shoot commonly mobile RNAs decreased under Pi deficiency, while the MI of four shoot‐to‐root and eight root‐to‐shoot common mobile RNAs increased in response to Pi deficiency (Figure S3, Supporting Information). These results indicate that besides the specific mobility of RNAs, the mobile abundance of common mobile RNAs also responds to Pi deficiency.

Most of these mobile RNAs were protein‐coding mRNAs (Figure S2G and Table S5, Supporting Information). We examined the functional annotations of the genes associated with these PSR‐specific mobile RNAs to evaluate their biological functions (Table S5, Supporting Information). Notably, most of the PSI‐specific shoot‐to‐root mobile RNAs were coding genes involved in photosynthesis‐related pathways, particularly in the case of the long‐term Pi starvation (NP7D)‐specific induced mobile RNAs (17/27 total annotated genes). This finding suggests non‐canonical functions for nucleus‐encoded photosynthesis‐related mRNAs.

In summary, these results indicate that Pi starvation induces a drastic change in long‐distance RNA transport and that these PSI‐specific shoot‐to‐root mobile RNAs are enriched in photosynthesis‐related pathways.

### 2.3 Pi Deficiency–Triggered Long‐Distance RNA Transport is not due to Expression Changes in the Donor Tissues

Because Pi deficiency alters the expression of thousands of PSR genes,^[^ ^36^ ^]^ we wondered whether the presence of RNAs in recipient tissues in response to Pi deficiency might simply reflect changes in the expression of genes in the donor tissues. To exclude an effect from heterografting, we compared the transcript abundance of predicted mobile RNAs in the donor tissues between heterografts and homografts. Notably, transcript levels were comparable between heterograft and homograft samples for these PSR‐mobile RNAs under +P and −P conditions (*R* = 0.968–0.990), indicating that the PSR‐induced mobility of identified transcripts is not caused by an increase in their abundance in the donor tissues due to heterografting (Figure S4, Supporting Information).

We then investigated the changes in gene expression between Pi‐sufficient and Pi‐deficient conditions. Consistent with previous studies, Pi deficiency induced or repressed the expression of thousands of genes (Figure 1H,I; Table S2, Supporting Information). However, the expression levels of most PSR‐mobile RNAs (88.2% of shoot‐to‐root and 96.7% of root‐to‐shoot mobile RNAs in NP3D compared to FP7D; 83.15% of shoot‐to‐root and 88.3% of root‐to‐shoot mobile RNAs in NP7D compared to FP7D) did not exhibit differential expression between Pi‐sufficient and ‐deficient conditions in the donor tissue (Figure 1H,I). Of note, these mobile RNAs correspond to highly expressed genes in donor tissues, consistent with a previous study^[^ ^8^ ^]^ (Figure S5 and Table S7, Supporting Information). These results suggest that the mobility of these transcripts is unlikely to be a result of expression changes caused by exposure to Pi deficiency in the donor tissue, although we cannot exclude the possibility of tissue‐specific expression changes for those mobile transcripts, such as in companion cells.

### 2.4 Long‐Distance Transport of PSR‐Specific mRNAs is Largely Mediated by PHRs

**Figure 2:**
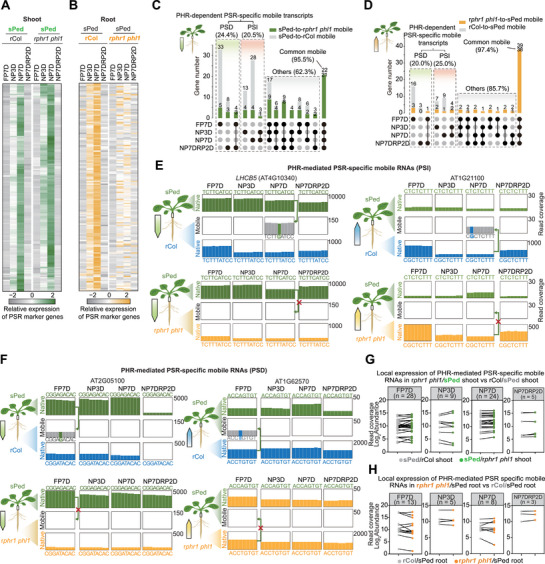
PSR‐specific mobile RNAs are largely mediated by PHRs via a function distinct from direct transcriptional regulation. A,B) Relative expression patterns of PSR marker genes in the A) shoots and B) roots of r*phr1 phl1*/sPed and rCol/sPed chimeric plants. Expression levels of PSR marker genes were substantially lower in the roots of r*phr1 phl1*/sPed compared to those in the roots of rCol/sPed. C,D) Diagrams showing that the mobility of PSR‐specific mobile RNAs from C) shoot‐to‐root and D) root‐to‐shoot is largely dependent on PHR function. Gray bars indicate the number of mobile RNAs that were identified in rCol/sPed but not in r*phr1 phl1*/sPed; green and orange bars indicate the number of shoot‐to‐root (green) and root‐to‐shoot (orange) mobile RNAs that were identified in both rCol/sPed and r*phr1 phl1*/sPed, respectively. The percentage = given in the plots represent the number of mobile transcripts in r*phr1 phl1*/sPed versus the number of mobile genes in rCol/sPed. E,F) Integrative Genome Viewer browser windows of read coverage for informative SNPs in the coding sequence of a E) PHR‐mediated PSI mobile mRNA (*LHCB5* and At1g21100) or F) PHR‐mediated PSD mobile mRNAs (At2g05100 and At1g62570). Each plot represents the cumulative read count from Col‐0 (blue bars), Ped‐0 (green bars), and *phr1 phl1* (orange bars). In mobile boxes, gray bars indicate identical nucleotides between Ped‐0 and Col‐0, and green and blue bars indicate the informative SNPs between Ped‐0 and Col‐0. Directions of the green, yellow, or blue arrows show the predicted transcript movement from shoot (sPed) to root (rCol or r*phr1 phl1*) or root (r*phr1 phl1* or rCol) to shoot (sPed). G,H) Local expression of PHR‐mediated PSR‐specific mobile RNAs is largely unchanged in r*phr1 phl1*/sPed compared to that in rCol/sPed in (G) shoots and H) roots. Gray dots indicate gene expression in rCol/sPed shoots or roots; green and orange dots indicate gene expression in r*phr1 phl1*/sPed shoots and r*phr1 phl1*/sPed roots, respectively.

Because PHR1 and its homologs are evolutionarily conserved core TFs in PSR signaling networks,^[^ ^34^, ^36^, ^48^, ^49^ ^]^ we asked whether the PHR‐mediated PSR signaling network regulates PSR long‐distance transport of RNA. To this end, we first created a *phr1 phl1* double mutant and confirmed the decreased expression of PSR genes in this double mutant under −P (Figure S6A–C, Supporting Information). PHL1 is a homolog of PHR1.^[^ ^36^ ^]^ With this *phr1 phl1* mutant, we produced heterografts between Ped‐0 shoots and *phr1 phl1* roots (hereafter as r*phr1 phl1*/sPed) and then extracted RNA from shoot and root samples for RNA‐seq. The three biological replicates for each sample showed excellent reproducibility, as seen by clustering and PCA (Figure S6D,E, Supporting Information). The expression of PSR marker genes^[^ ^50^ ^]^ was much less induced in the roots of r*phr1 phl1*/sPed chimeric plants, but not in their shoots (**Figure** **2**A,B; Figure S6F and Table S3, Supporting Information).

We identified mobile RNAs in r*phr1 phl1*/sPed plants based on our custom pipeline (Figures S1D and S7A,B and Table S5, Supporting Information). The loss of PHR function in roots eliminated 34 out of 94 root‐to‐shoot mobile RNAs. Importantly, most of these RNAs were PSR‐specific mobile RNAs (31/34) (Figure 2D). Notably, 90 out of 164 shoot‐to‐root mobile RNAs, which were mostly PSR specific (69/90), were no longer mobile when *phr1 phl1* was used as the rootstock (Figure 2C; Table S5, Supporting Information). Visualization of read counts via the Integrative Genomic Viewer for informative SNPs within predicted mobile RNAs for *LHCB5* (AT4G10340), *PORB* (AT4G27440), AT2G05100, AT4G14400, AT1G21100, and AT1G62570 confirmed the loss of translocation of these mRNAs (Figure 2E,F; Figure S7C,D, Supporting Information). Notably, decreased PSR signaling in roots impaired both shoot‐to‐root and root‐to‐shoot transport of PSR‐specific RNAs, indicating that PSR‐specific RNA mobility entails PSR signaling in both donor and recipient tissues (Figure 2A–D).

We explored whether the changes in PSR‐specific RNA mobility in r*phr1 phl1*/sPed heterografted plants resulted from altered expression by comparing the genes represented by mobile RNAs to published chromatin immunoprecipitation followed by sequencing (ChIP‐seq) datasets for PHR1.^[^ ^50^ ^]^ Fewer than 16 of these genes were direct targets of PHR1 (Figure S7A,B and Table S5, Supporting Information). Next, we compared the abundance of mobile RNAs between rCol/sPed and r*phr1 phl1*/sPed heterografts. The mutation of *PHR*s did not change the expression levels of most PHR‐mediated mobile RNAs in either shoots or roots under Pi sufficiency or Pi deficiency conditions (Figure 2G,H; Table S7, Supporting Information). The loss of RNA movement without concomitant changes in expression levels indicates that the transport of these PSR‐specific mobile RNAs is largely mediated by PHRs via a function distinct from direct transcriptional regulation.

### 2.5 The PHR‐Mediated PSR‐Specific Mobile mRNAs are not Translated in Recipient Tissues

**Figure 3:**
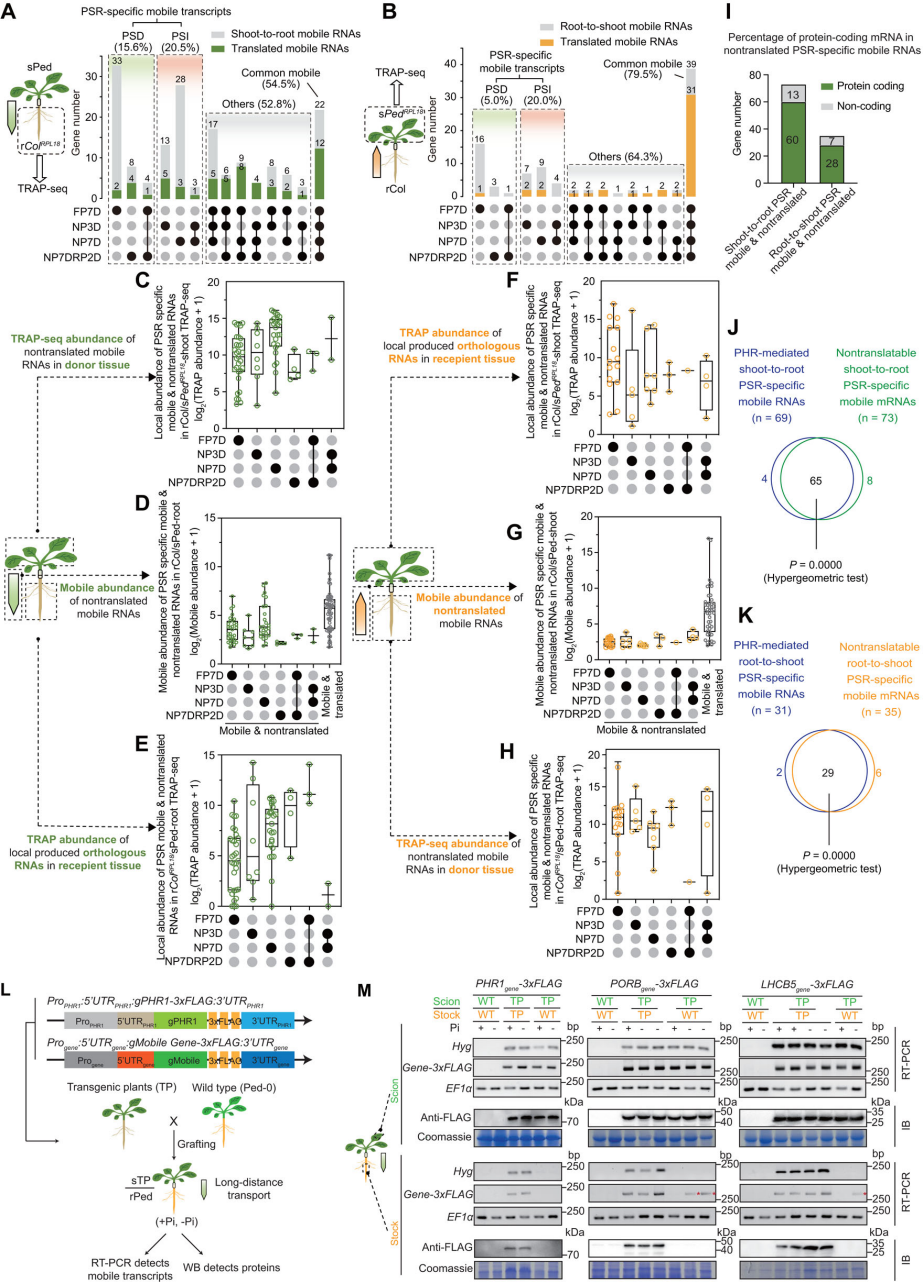
PHR‐mediated PSR‐specific mobile mRNAs are not translated in recipient tissues. A,B) Diagrams showing that most PSR‐specific mobile RNAs, from A) shoot‐to‐root or B) root‐to‐shoot, are not translated in recipient tissues. Gray bars indicate the number of mobile genes found in the RNA‐seq data but not in the TRAP‐seq data of heterograft samples, suggestive of untranslated mobile RNAs. Green and orange bars indicate the numbers of mobile transcripts found in both RNA‐seq and TRAP‐seq data of heterograft samples, suggestive of translated mobile RNAs. The percentage = number of mobile & translated transcripts in TRAP‐seq vs number of mobile genes in RNA‐seq. C,H) RNA abundance of the locally expressed RNAs in the TRAP‐seq data for C) shoot‐to‐root and H) root‐to‐shoot PSR‐specific mobile untranslated RNAs in donor tissue, indicating that they can be translated before their long‐distance movement. E,F) Abundance of the corresponding locally expressed RNAs in the TRAP‐seq data for E) shoot‐to‐root and F) root‐to‐shoot PSR‐specific mobile RNAs in recipient tissue showing that most are translated. D,G) Mobile abundance of untranslated D) shoot‐to‐root and G) root‐to‐shoot PSR‐specific mobile RNAs is comparable to that of translated mobile transcripts. The lines and box edges indicate the median and quartiles, respectively. I) Bar charts indicating that most shoot‐to‐root and root‐to‐shoot PSR‐specific mobile untranslated RNAs are annotated as protein‐coding transcripts. J,K) Venn diagrams indicating that most PSR‐specific mobile untranslated mRNAs are PHR dependent in (J) shoot‐to‐root and K) root‐to‐shoot mobile mRNAs. *P*‐values were calculated using the hypergeometric test. L) Diagram of the constructs consisting of the coding region of a mobile or nonmobile (*PHR1*) gene, cloned in‐frame and upstream of the sequence encoding a 3xFLAG tag and driven by the endogenous promoter used for grafting tests between transgenic and wild‐type plants. M, RT‐PCR, and immunoblot (IB) assays confirming PSR‐specific long‐distance RNA transport and non‐translation in recipient tissues of the chimeric transcripts *PORB‐3xFLAG* and *LHCB5‐3xFLAG* as examples of PHR‐mediated PSR‐specific mobile mRNAs. *PHR1‐3xFLAG* and the hygromycin resistance gene (*Hyg*) served as nonmobile RNA controls.

Because most mobile RNAs identified here were annotated as protein‐coding mRNAs (Figure S2G and Table S5, Supporting Information), we asked whether these RNAs would be translated in recipient tissues. Accordingly, we created transgenic plants harboring a construct consisting of the Cauliflower mosaic virus 35S promoter driving a sequence encoding RIBOSOMAL PROTEIN L18 (RPL18) tagged with 3×FLAG in the Col‐0 and Ped‐0 backgrounds (*35S:3×FLAG‐RPL18* in Ped‐0, referred to as *Ped^RPL18^* and *35S:3×FLAG‐RPL18* in Col‐0, referred to as *Col^RPL18^*) (Figure S8A, Supporting Information). We then conducted TRAP‐seq^[^ ^51^ ^]^ on reciprocal heterografts to evaluate the translation landscape of these mobile mRNAs under Pi sufficiency or deficiency conditions (**Figure** **3**A,B; Figure S8A, Supporting Information). We affinity‐purified ribosome–RNA complexes using an anti‐FLAG antibody and subjected the RNAs to strand‐specific RNA‐seq (Figure S8B and Tables S8 and S9, Supporting Information). We identified translated and mobile mRNAs based on the presence of SNPs with our pipeline (Figure 3A,B; Figure S1D, Supporting Information). Using three biological replicates per sample (>25 plants per replicate), the TRAP‐seq data showed high reproducibility among replicates (Figure S8C–F and Tables S10, Supporting Information). The abundance of PSR transcripts bound to ribosomes also showed the expected pattern in the TRAP‐seq data, indicating the efficacy of our growth conditions to induce Pi deficiency (Figure S8G,H, Supporting Information).

Following our pipeline (Figure S1D, Supporting Information), we identified 57 shoot‐to‐root and 45 root‐to‐shoot translated mobile RNAs (Figure 3A,B; Table S11, Supporting Information). About half of the mobile RNAs were translated in recipient tissues, most of which were common mobile RNAs (54.5% of shoot‐to‐root and 79.5% of root‐to‐shoot) among the four Pi treatments, regardless of the direction of movement (Figure 3A,B; Table S11, Supporting Information). To assess Pi deficiency–mediated alterations in the translation of mobile mRNAs, we calculated their translational efficiency (TE = RNA abundance in TRAP‐seq data/abundance in RNA‐seq) during Pi deficiency. Seven out of 12 translated common shoot‐to‐root mobile mRNAs had a higher TE in recipient tissues under at least one Pi deficiency treatment compared to Pi sufficiency (Figure S9, Supporting Information). Among 31 translated root‐to‐shoot common mobile mRNAs, the TE of 11 mobile mRNAs was lower while the TE of nine mobile mRNAs was higher in response to Pi deficiency (Figure S9, Supporting Information). The TE patterns of these mobile mRNAs were largely different under Pi deficiency conditions from those of their locally expressed counterpart genes in recipient tissues (Figure S9 and Table S12, Supporting Information). The change in TE of most common mobile mRNAs under Pi deficiency was not observed for their locally expressed counterpart genes in recipient tissues. This finding indicates that the translational regulation of mobile mRNAs is independent from the translation of their corresponding local mRNAs. There may be unknown factors involved in long‐distance mRNA transport that contribute to this differential regulation.

Of note, most of the PSR‐specific mobile RNAs (82.0% of shoot‐to‐root and 87.5% of root‐to‐shoot) were not detected in our TRAP‐seq data, suggesting that they may not be translated in recipient tissues (Figure 3A,B; Table S11, Supporting Information). To rule out low abundance of these RNAs as a reason for their absence in our TRAP‐seq dataset, we compared the abundance of translated and nontranslated mobile RNAs in the RNA‐seq data. Notably, the abundance of these nontranslated mobile RNAs was comparable to that of translated mobile RNAs, excluding the possibility that their absence from the TRAP‐seq data was caused by low transcript abundance (Figure 3D,G). We also can detect the mobile transcripts in TRAP‐seq input samples, indicating that the absence of these transcripts in IP reflects their lack of ribosome binding (Figure S8I, Supporting Information). We then wondered whether those nontranslated mobile RNAs were translatable but not translated or lost the ability to be translated during transport. An analysis of the TRAP‐seq data for local and mobile RNAs in donor and recipient tissues indicated that almost all local RNAs corresponding to these nontranslated mobile RNAs are translated in both donor and recipient tissues (Figure 3C,E,F,H; Table S9, Supporting Information). This result confirms that the translational regulation of mobile mRNAs in recipient tissues is independent of the translation of their corresponding local mRNAs. Moreover, these nontranslated mobile mRNAs (89.0% of shoot‐to‐root and 82.9% of root‐to‐shoot) were mostly protein‐coding RNAs (Figure 3I) and significantly overlapped with the PHR‐mediated PSR‐specific mobile mRNAs (94.2% of shoot‐to‐root and 93.5% of root‐to‐shoot, *P* = 0, hypergeometric test), regardless of the direction of movement (Figure 3J,K).

To confirm that PSR‐specific mobile transcripts are not translated in the recipient tissue, we selected two candidate genes (*PORB* and *LHCB5*) whose mRNAs are PHR‐mediated mobile in response to long‐term Pi deficiency (NP7D) from shoot to root. We generated transgenic plants with the endogenous promoter driving the expression of each genomic coding region cloned in‐frame and upstream of the sequence for a 3xFLAG, followed by the endogenous 3′ UTR of that gene, yielding the constructs *Pro_PORB_:5*′*UTR_PORB_:gPORB‐3xFLAG:3*′*UTR_PORB_* and *Pro_LHCB5_:5*′*UTR_LHCB5_:gLHCB5‐3xFLAG:3*′*UTR_LHCB5_*. We also developed transgenic plants harboring a *Pro_PHR1_:5*′*UTR_PHR1_:gPHR1‐3xFLAG:3*′*UTR_PHR1_* construct as a control for a nonmobile mRNA. We grafted transgenic plants of the T2 generation in the Col‐0 background with Ped‐0 (Ped‐root/transgenic plants‐shoot [rPed/sTP]) and exposed the grafts to Pi sufficiency or long‐term Pi starvation conditions (Figure 3L). We detected the corresponding *Gene‐3xFLAG* chimeric transcripts and their Gene‐3xFLAG proteins in the scion (shoot, donor tissue) of all grafted materials (Figure 3M). Notably, we only detected *PORB‐3xFLAG* and *LHCB5‐3xFLAG* chimeric transcripts in the stock (root, recipient tissue) of grafted materials under Pi‐deficient but not Pi‐sufficient conditions, confirming PSI long‐distance RNA transport. However, we did not detect PORB‐3xFLAG or LHCB5‐3xFLAG proteins in the stock (root, recipient tissue) of grafted materials under either Pi‐sufficient or ‐deficient conditions (Figure 3M). These results confirmed the PSI long‐distance transport and lack of translation for these PSR‐specific mobile RNAs in the recipient tissues.

Our results suggest an independent Pi deficiency–mediated translational regulation of mobile mRNAs in recipient tissues that differs from the translation of their corresponding locally expressed mRNAs. Furthermore, our results indicate that PHR‐mediated PSR‐specific mobile mRNAs may have functions independent of that of their encoding proteins in recipient tissues.

### 2.6 PHR‐Mediated PSR‐Specific Mobile mRNAs Have a Less Structured 5′ UTR

**Figure 4:**
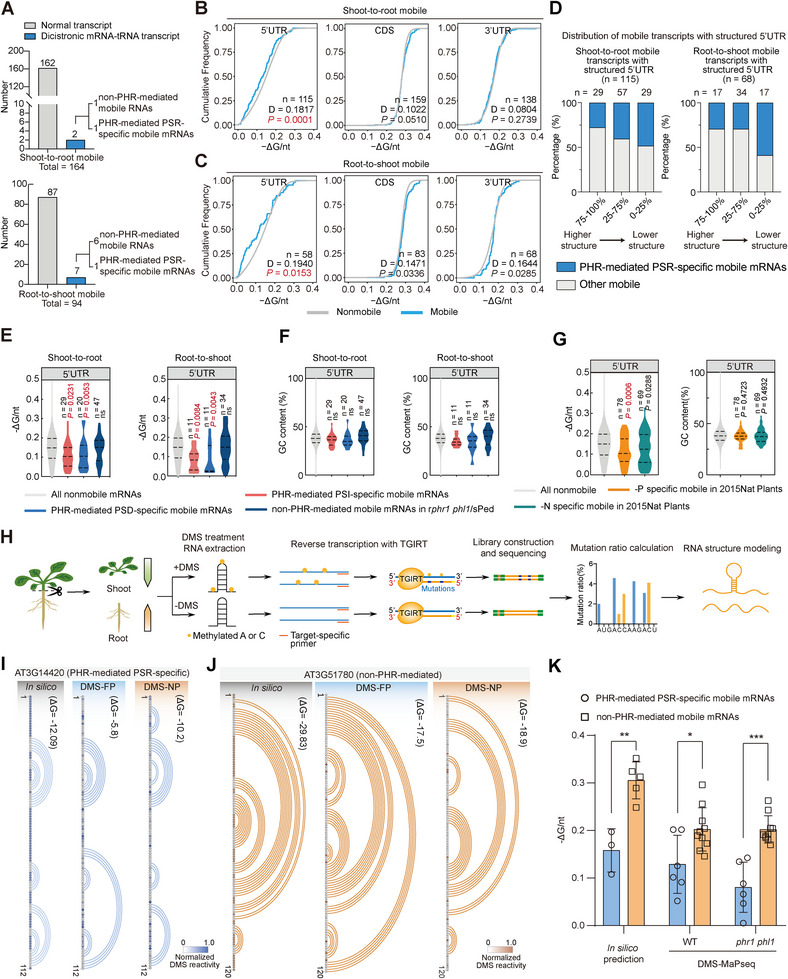
PHR‐mediated PSR‐specific mobile mRNAs have a less structured 5′ UTR. A) Bar chart showing the number of mobile RNAs (2/164 of shoot‐to‐root, 7/94 of root‐to‐shoot) that are dicistronic mRNA‐tRNA transcripts, two of which are PHR‐mediated PSR‐specific mobile mRNAs. B,C) Cumulative distributions of –ΔG/nt for the 5′ untranslated region (5′ UTR), coding sequence (CDS), and 3′ UTR for all mobile and nonmobile RNAs from B) shoot‐to‐root and C) root‐to‐shoot. The RSS of the 5′ UTR is less‐structured in mobile RNAs versus in nonmobile RNAs, indicated by the lower –ΔG/nt values. D‐values represent the maximal separation between these two populations. *P*‐values were calculated using the Kolmogorov‐Smirnov test. n values represent the number of genes used for analysis. D) Bar charts indicating that the PHR‐mediated PSR‐specific mobile RNAs are mainly enriched in the group with the least structured 5′ UTRs (0–25%). E) Violin plots showing that the 5′ UTRs of PHR‐mediated PSI‐ and PSD‐specific mobile mRNAs harbor a more unfolded RSS than either nonmobile or non‐PHR‐mediated mobile mRNAs. Lower –ΔG/nt values represent a more unfolded RSS. F) Violin plots showing slight differences in the GC content of 5′ UTRs for PHR‐mediated PSI‐ and PSD‐specific mobile mRNAs from either non‐PHR‐mediated mobile mRNAs or nonmobile mRNAs. G) Violin plots showing the –ΔG/nt (left) and GC content (right) of 5′ UTRs among −P‐specific mobile (*n* = 78), −N‐specific mobile (*n* = 69), and nonmobile RNAs published in the 2015Nat Plants^[^ ^8^ ^]^ study. Published −P‐specific mobile RNAs also harbor more unfolded 5′ UTR than that of either −N‐specific mobile RNAs or nonmobile RNAs. H) Workflow of target‐specific DMS‐MaPseq. I and J, Arch diagrams showing the RSS of 5′ UTRs for I) PHR‐mediated PSR‐specific mobile mRNAs (AT3G14420) and J) non‐PHR‐mediated mobile mRNAs (AT3G51780) with in silico prediction or based on target‐specific DMS‐MaPseq Data. K, Bar plots show that the −ΔG/nt of 5’UTR RSS of tested PHR‐mediated PSR‐specific mobile mRNAs is significantly lower than that of tested non‐PHR‐mediated mobile mRNAs in both in silico prediction data and DMS‐MaPseq data. *, *P* < 0.05, **, *P* < 0.01, ***, *P* < 0.001; two‐tailed Student's *t*‐test. The values are means ± standard deviation (SD). In E‐G, *P*‐values were calculated using the Wilcoxon test. n values represent the number of genes used for analysis. All data for analysis are provided in Table S13–S18 (Supporting Information).

The above results raised the question as to whether these PHR‐mediated PSR‐specific mobile mRNAs exhibit distinct RNA structural features. A tRNA‐like structure (TLS) was reported in some mobile mRNAs.^[^ ^12^ ^]^ Consistent with a previous study,^[^ ^12^ ^]^ we determined that a small proportion of our identified mobile mRNAs (≈3.5%) corresponds to dicistronic mRNA:tRNA transcripts (**Figure** **4**A; Table S5, Supporting Information). Moreover, only two of the PHR‐mediated PSR‐specific mobile mRNAs had a TLS (Figure 4A), suggesting that a potentially unknown RNA structural feature regulates RNA mobility in response to Pi deficiency.

Next, we characterized the RNA secondary structure (RSS) of mobile mRNAs using their length‐normalized minimum thermodynamic free energy (−ΔG/nt).^[^ ^52^ ^]^ The −ΔG/nt values calculated by in silico prediction were comparable to the values calculated by dimethyl sulfate (DMS)‐guided in vivo RNA structure profiling.^[^ ^52^ ^]^ A higher −ΔG/nt value indicates more folding in the RSS, and a lower value indicates less folding. The shoot‐to‐root and root‐to‐shoot mobile mRNAs exhibited a lower −ΔG/nt value in their 5′ UTR than nonmobile mRNAs (Figure 4B,C; Tables S13–S15, Supporting Information), indicating a less structured RSS in the 5′ UTR of mobile mRNAs. Importantly, this difference in RSS was not due to the GC content, which was not significantly different between mobile and nonmobile mRNAs (Figure S10A and Tables S13 and S16, Supporting Information). Moreover, the root‐to‐shoot mobile mRNAs, but not the shoot‐to‐root mobile mRNAs, exhibited slightly more folding (higher −ΔG/nt) in the coding sequence and 3′ UTR (Figure 4C; Table S14, Supporting Information). These features were also not due to differences in GC content (Figure S10A and Table S16, Supporting Information). Besides the RSS, the 5′ UTRs of mobile mRNAs were in general shorter than those of nonmobile mRNAs, both for shoot‐to‐root and root‐to‐shoot mobile RNAs (Figure S10B and Table S13, Supporting Information). These results suggest that an intrinsic RNA structure, particularly the RSS of 5′ UTR, at the mobile mRNAs.

We classified all mobile mRNAs into three categories according to their −ΔG/nt: mobile RNAs with the highest −ΔG/nt in the 5′ UTR (top 25%, highly structured 5′ UTRs), those with the lowest −ΔG/nt in the 5′ UTR (bottom 25%, poorly structured 5′ UTRs), and all other mobile RNAs (Figure 4D). We observed that PHR‐mediated PSR‐specific mobile mRNAs are enriched in the bottom 25% of RNAs based on their −ΔG/nt (Figure 4D). We independently divided mobile mRNAs into PHR‐mediated PSR‐specific mobile mRNAs and non‐PHR‐mediated mobile mRNAs. Compared to nonmobile mRNAs and non‐PHR‐mediated mobile mRNAs, PHR‐mediated PSR‐specific mobile mRNAs exhibited significantly less structured 5′ UTRs (lower −ΔG/nt) (Figure 4E). This scenario occurred in both shoot‐to‐root and root‐to‐shoot PHR‐mediated PSI‐specific and PSD‐specific mobile mRNAs (Figure 4E; Table S13, Supporting Information). However, we also noticed marginal differences in the 3′ UTR and coding sequences (Figure S10C, Supporting Information). To confirm these results, we analyzed the RSS of nutrient deficiency–induced mobile transcripts from previous studies (referred to collectively as 2015 Nat. Plants^[^ ^8^ ^]^). In line with our results, the −Pi‐specific mobile transcripts from the 2015Nat. Plants study also had more unfolded 5′ UTRs compared to either nonmobile transcripts or nitrogen‐deficient (−N)‐specific mobile transcripts (Figure 4G; Figure S10D and Table S17, Supporting Information). As with our data, the less structured 5′ UTRs of −Pi‐specific mobile mRNAs was not related to the GC content (Figure 4F,G; Table S18, Supporting Information). These results suggest that a less structured 5′ UTR may be a specific RNA structural feature for PSR‐specific mobile mRNAs.

To further validate this RSS feature, we performed target‐specific DMS‐MaPseq (DMS mutational profiling with sequencing)^[^ ^53^, ^54^ ^]^ to profile the in vivo RSS in the 5’UTR of mobile mRNAs in the donor tissues in WT and *phr1 phl1* plants under Pi‐sufficiency (FP) and Pi‐deficiency (NP) conditions (see Experimental Section). The in vivo RSS data of 18S ribosomal RNA (rRNA) validated the fidelity and high‐quality of our DMS‐MaPseq data (Figure S11, Supporting Information). Next, we obtained reliable DMS‐MaPseq data for three PHR‐mediated PSR‐specific and five non‐PHR‐mediated mobile mRNAs (see Experimental Section). We modeled the RSS of 5’UTRs based on the DMS‐MaPseq data using RNAstructure^[^ ^55^ ^]^ or in silico prediction using RNAfold.^[^ ^56^ ^]^ The ΔG values were similar between in silico predicted RSS and DMS‐MaPseq data‐based RSS (Figure 4I,J; Figures S12 and S13, Supporting Information), confirming the fidelity of our in silico RSS analysis (Figure 4B–G). Notably, both in silico predicted and DMS‐MaPseq‐based RSS in the WT exhibited less‐structured 5’UTRs for PHR‐mediated PSR‐specific mobile mRNAs, whereas the RSS of non‐PHR‐mediated mobile mRNAs exhibited complicated stem‐loop structures (Figure 4I,J; Figures S12 and S13, Supporting Information). This scenario occurred under both FP and NP conditions (Figure 4I,J; Figures S12 and S13, Supporting Information). Consistently, the overall −ΔG/nt of profiled 5’UTRs of PHR‐mediated PSR‐specific mobile mRNAs was significantly lower than that of non‐PHR‐mediated mobile mRNAs (Figure 4K), while the overall −ΔG/nt of profiled 5’UTRs was unchanged between FP and NP conditions in either PHR‐mediated or non‐PHR‐mediated mobile mRNAs (Figure S14, Supporting Information). We further compared the RSS of profiled 5’UTR between WT and *phr1 phl1* plants. Although the modeled RSS and corresponding ΔG values were not identical in many tested transcripts between WT and *phr1 phl1* plants, the overall −ΔG/nt of profiled 5’UTRs did not significantly differ between the WT and *phr1 phl1* in either PHR‐mediated or non‐PHR‐mediated mobile mRNAs under FP or NP conditions (Figures S12–S14, Supporting Information).

In summary, our target‐specific DMS‐MaPseq results confirmed the less‐structured 5’UTRs of PHR‐mediated PSR mobile mRNAs. Our results also showed that this RSS feature is not altered in WT and *phr1 phl1* under either FP or NP conditions, suggesting an intrinsic RNA structural feature.

### 2.7 A Less‐Structured 5’UTR Alone is not Essential for PSR Long‐Distance mRNA Transport

To test whether a less structured 5′ UTR is essential for PSR long‐distance mRNA transport, we swapped the 5′ UTRs between the nonmobile mRNA *PHR1* (ΔG of *PHR1* 5’UTR_PHR1_ = −43.80) and two PSR‐specific mobile mRNAs (ΔG of *LHCB5* 5′ UTR = −27.30 and ΔG of *PORB* 5′ UTR = −5.50) (Figure S15A, Supporting Information). This 5’UTR exchange generated four transgenic constructs: *Pro_PHR1_:5*′*UTR_LHCB5_:gPHR1‐3xFLAG:3*′*UTR_PHR1_*, *Pro_PHR1_:5*′*UTR_PORB_:gPHR1‐3xFLAG:3*′*UTR_PHR1_*, *Pro_LHCB5_:5*′*UTR_PHR1_:gLHCB5‐3xFLAG:3*′*UTR_LHCB5_* and *Pro_PORB_:5*′*UTR_PHR1_:gPORB‐3xFLAG:3*′*UTR_PORB_* (Figure S15B,D, Supporting Information). We then obtained T2 transgenic plants harboring these constructs (designated as *5’UTR_Gene_‐gPHR1* and *5’UTR_PHR1_‐gGene*), along with two control transgenic lines: *Pro_RORB_:5*′*UTR_PORB_:gPORB‐3xFLAG:3*′*UTR_PORB_* and *Pro_LHCB5_:5*′*UTR_LHCB5_:gLHCB5‐3xFLAG:3*′*UTR_LHCB5_*. These transgenic plants were grafted with Ped‐0 wild‐type root and subjected to either FP or NP treatment for 7 days.

In rWT/s*5’UTR_Gene_‐gPHR1* grafted plants, we detected the *PHR1‐3xFLAG* transcripts in shoots (donor tissue), but not in roots (recipient tissue) under both FP and NP conditions (Figure S15C, Supporting Information). Meanwhile, in rWT/s*5’UTR_PHR1_‐gGene* grafted plants, the PSR mobility of *PORB‐3xFLAG* and *LHCB5‐3xFLAG* transcripts was not altered under NP conditions when the self‐5’UTR was replaced by the *PHR1* 5’UTR (Figure S15E, Supporting Information). These results suggest that a less structured 5′ UTR alone is not essential for PSR long‐distance mRNA transport.

## 3 Discussion

**Figure 5:**
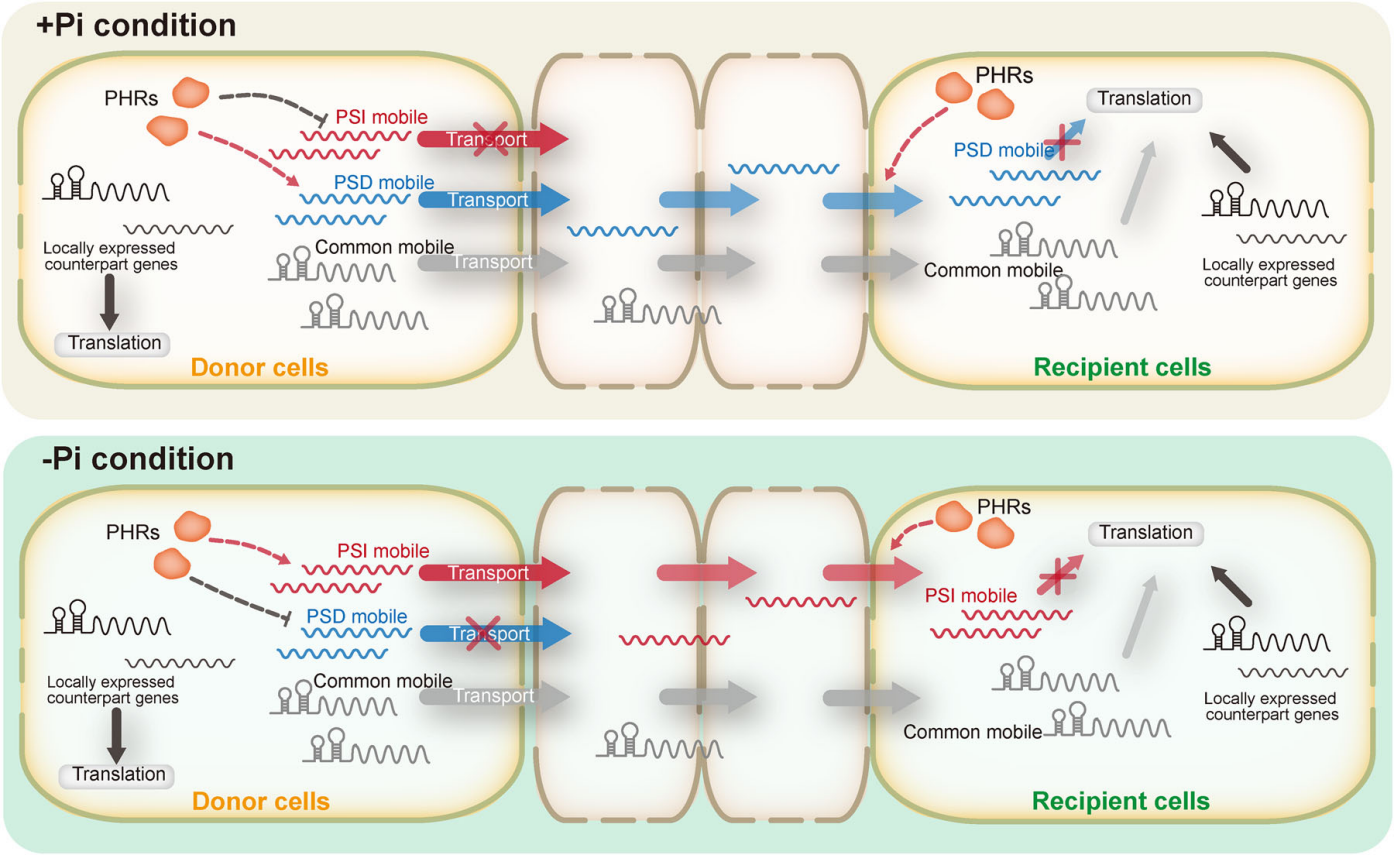
A proposed model showing the PHR‐mediated PSR‐specific mobile mRNAs are untranslated transcripts in recipient tissues after their long‐distance transport. Under normal conditions, common and PSD mobile RNAs can be transported over a long distance from donor cells to recipient cells. Under Pi deficiency conditions, the mobility of PSD mobile RNAs decreases, whereas that of PSI mobile RNAs rises. The mobility of PSD and PSI mobile RNAs is dependent on PHR function but not via direct transcriptional regulation. Those PSD and PSI mobile RNAs may have a function independent of their encoding protein in recipient tissues, although they can be translated in donor tissues.

The response of plants to Pi deficiency involves systemic PSR signaling that integrates information about Pi levels from different plant tissues.^[^ ^27^ ^]^ Long‐distance RNA transport is one of the methods for communication between tissues,^[^ ^4^, ^5^, ^27^ ^]^ as was reported for Pi deficiency.^[^ ^7^, ^8^ ^]^ However, the function, regulatory mechanism(s), and features of these PSR‐mobile RNAs are still poorly characterized. In this work, we defined the global landscape of mobile RNAs that are transported over long distances (between shoots and roots) under Pi sufficiency and Pi deficiency conditions and identified ≈100 PSR‐specific RNAs that were mobile between shoots and roots (Figure 1A–G). But the expression levels of these PSR‐specific mobile transcripts were comparable between normal and Pi starvation conditions in both Col‐0 and Ped‐0 background (Figure 1H,I; Figure S4, Supporting Information). Using heterografts between *phr1 phl1* and wild‐type seedlings, we determined that the PHR proteins PHR1 and PHL1 were required for the transport of these PSR‐specific mobile RNAs (Figure 2C–F) but did not require direct transcriptional regulation of the loci from which these mobile RNAs are expressed (Figure 2G,H). Translatome profiling by TRAP‐seq and transgenic approach surprisingly indicated that these PHR‐mediated PSR‐specific mobile mRNAs, but not other mobile mRNAs, were not translated in recipient tissues, although they are bona fide protein‐coding sequences actively translated in donor tissues (Figure 3). An RNA structure analysis showed that these PHR‐mediated PSR‐specific mobile mRNAs harbored a less structured 5′ UTR, a special RSS feature we discovered in Pi deficiency‐related mobile mRNAs (Figure 4). Our results demonstrate that PHR proteins regulate PSR‐induced long‐distance transport of mRNA and that these PSR‐specific mRNAs may play protein‐independent functions in recipient tissues (**Figure** **5**).

PHRs have been shown to be key TFs that regulate the transcriptional response to Pi deficiency.^[^ ^34^, ^36^ ^]^ Our results showed that PHRs also regulate PSR‐specific long‐distance mRNA transport (Figure 2). Heterografts between wild‐type and *phr1 phl1* mutant seedlings revealed that the mobility of most PSR‐specific mobile RNAs was dependent on PHRs (Figure 2C,D). However, PHRs did not affect the expression levels of these PSR‐specific mobile RNAs in the donor tissues (Figure 2G,H). This mechanism is different from the dose‐dependent long‐distance transport of the PSI microRNA 399 (miR399), where PHR1 directly induces the expression of the *MIR399* loci to trigger long‐distance transport.^[^ ^33^, ^41^, ^42^ ^]^ Thus, distinct types of regulation exist for the long‐distance transport of mRNA or miRNA. In addition, heterografted r*phr1 phl1*/sPed plants exhibited diminished PSR signaling in roots and normal PSR signaling in shoots, but both shoot‐to‐root and root‐to‐shoot PSR‐specific RNA transport were impeded, indicating that PSR‐specific mRNA mobility involves PSR signaling in both recipient and donor tissues simultaneously (Figure 2A–D). Such a scenario suggests that PSR‐specific mRNA transport requires unknown PHR‐regulated *trans* factors involved in sending and receiving mobile mRNAs. The mechanism by which PHRs regulate mRNA mobility remains uncertain. PHRs might indirectly regulate PSR‐induced mRNA mobility via either downstream PSR signaling or some unknown pathways, such as through influencing the expression of protein‐coding genes that mediate the transport of mobile mRNAs. However, this does not rule out the possibility of PHRs exerting a direct regulatory role on PSR mobile mRNAs. Notably, recent study had reported that many human TFs possess RNA‐binding capabilities.^[^ ^57^ ^]^ To date, however, there is no experimental evidence to validate whether members of the PHR family also exhibit such RNA‐binding activity. Therefore, investigating the specific manner in which PHRs influence mRNA mobility represents a critical direction for future research.

TRAP‐seq and transgenic plants demonstrated that PHR‐mediated PSR‐specific mobile mRNAs were not translated into proteins in recipient tissues, indicative of a noncoding function for these mobile transcripts. Previous studies have demonstrated that mobile mRNAs can be translated to proteins to perform appropriate functions in recipient tissues.^[^ ^8^, ^12^, ^14^ ^]^ The mobile mRNAs were always analyzed and clustered using Gene Ontology (GO) analysis based on their protein‐coding functions in previous studies.^[^ ^7^, ^8^, ^58^, ^59^ ^]^ However, it is unclear whether all mobile mRNAs can be translated to produce functional proteins. We performed TRAP‐seq with heterografting to allow a genome‐wide investigation of mobile mRNA translation under normal and Pi starvation conditions. Notably, non‐PSR‐mobile mRNAs were translated, but PSR‐specific mobile mRNAs were mostly not translated in recipient tissues (Figure 3). Furthermore, these PSR‐specific mobile mRNAs were translated in donor tissues, as were their corresponding locally‐expressed mRNAs in recipient tissues (Figure 3C–H). These results indicate that these PSR‐specific mobile mRNAs have lost translational ability after long‐distance transport and may perform a protein‐independent function in the recipient tissue. We hypothesize the following possibilities regarding this phenomenon: 1) PSR‐specific mobile transcripts may not be full‐length, as the SNP‐dependent mobile RNA identification method cannot distinguish between full‐length and partial transcripts; 2) the PSR‐specific mobile transcripts may form certain RSSs and/or associate with *trans* factors, inhibiting their translation in recipient tissues; and 3) the PSR‐specific mobile transcripts may have RNA modifications such as *N^6^*‐methyladenosine (m^6^A)^[^ ^19^, ^60^ ^]^ or m^5^C that may eliminate their translation ability.^[^ ^14^, ^19^, ^61^ ^]^ mRNAs perform functions as long noncoding transcripts under certain conditions. For instance, besides its protein‐coding function, human *p53* mRNA can directly bind to murine double minute 2 (MDM2) and influence its E3 ligase function.^[^ ^62^ ^]^ Our results indicate that PSR‐specific mobile mRNAs function independently of the function of their encoded protein to participate in the Pi deficiency response. Moreover, most PSI‐specific mobile mRNAs are nuclear protein‐coding genes involved in photosynthesis‐related pathways (Table S5, Supporting Information), indicating an unknown protein‐independent non‐canonical function for these photosynthesis‐related mRNAs after PSI long‐distance RNA transport from shoot to root.

RNA structures endow RNA with multiple functions including long‐distance transport.^[^ ^63^, ^64^ ^]^ A TLS was found in some mobile mRNAs,^[^ ^12^ ^]^ but rarely in the PSR‐specific mobile mRNAs identified in this study (Figure 4A). Through in silico RNA structure analysis and in vivo RSS profiling by DMS‐MaPseq, we discovered that a less structured 5′ UTR is one of the RSS features of PSR‐specific mobile mRNAs in both our data and other published data (Figure 4; Figures S10–S14, Supporting Information). Notably, this structural feature did not stem from different GC content (Figure 4F,G). It was reported that the UTRs of *StBEL5* ^[^ ^15^ ^]^ and *AtGAI* ^[^ ^21^ ^]^ are involved in mediating their long‐distance transport in potato (*Solanum tuberosum*) and *Arabidopsis*, respectively. But the results of the 5′ UTR swapping experiment indicate that a less structured 5′ UTR alone is not essential for PSR RNA mobility (Figure S15, Supporting Information). It implied a complex regulation in the long‐distance mRNA transport in response to Pi starvation.

In summary, our study combined mRNA mobileome, translatome, RNA structure analysis, and transgenic plants to decipher long‐distance mRNA transport triggered by Pi deficiency. We discovered that PHR‐mediated PSR‐specific mobile mRNAs are not translated in recipient tissues. This finding provides insights into the prevalence of stress‐induced long‐distance mRNA transport and suggests avenues for future research to better understand the protein‐independent functions of mobile mRNAs in response to Pi‐deficiency in plants.

## 4 Experimental Section

### Plant Materials and Growth Conditions

Seeds of the *Arabidopsis* (*Arabidopsis thaliana*) accessions Col‐0 and Pedriza‐0 (Ped) (CS76415) and the *phr1* (SALK_067629C) and *phl1* (CS832612) accessions were obtained from the *Arabidopsis* Biological Resource Center (https://abrc.osu.edu/).

Seeds were surface‐sterilized in 70% (v/v) ethanol for 30 s and then placed into a sodium hypochlorite solution (50% bleach/50% water/0.05% Tween, v/v/v) for 12 min with vigorous vortexing every 2 min. After four washes with sterile water, seeds were placed in the dark at 4 °C for 3 days. The seeds were then placed onto MS medium (1.9 g L^−1^ KNO_3_, 1.65 g L^−1^ NH_4_NO_3_, 0.37 g L^−1^ MgSO_4_•7H_2_O, 0.17 g L^−1^ KH_2_PO_4_, 0.44 g L^−1^ CaCl_2_•2H_2_O, 0.82 g L^−1^ K_2_SO_4_, 0.83 mg L^−1^ KI, 3.1 mg L^−1^ H_3_BO_3_, 0.25 mg L^−1^ MnSO_4_•4H_2_O, 8.6 mg L^−1^ ZnSO_4_•7H_2_O, 0.025 mg L^−1^ Na_2_MoO_4_•2H_2_O, 0.025 mg L^−1^ CuSO_4_•5H_2_O, 0.025 mg L^−1^ CoCl_2_•6H_2_O, 27.8 mg L^−1^ FeSO_4_•7H_2_O, 3.73 mg L^−1^ Na_2_EDTA, 0.1 mg L^−1^ inositol, 0.002 mg L^−1^ glycine, 0.001 mg L^−1^ vitamin B1 (VB1), 0.0005 mg L^−1^ VB6, and 0.0005 mg L^−1^ VB5) supplemented with 1% (w/v) sucrose and 0.05% (w/v) MES, pH 5.7 and solidified with 0.75% (w/v) agarose. Seeds were germinated by arranging the plates vertically in the dark for 2 days, and then moved to a growth chamber at 22 °C with a controlled 12‐h/12‐h (light/dark) photoperiod for 5 additional days.

### Construction of the pCAMBIA1300‐Pro_gene_:5′UTR_gene_:gGene‐3xFLAG:3′UTR_gene_ Vectors

The *Pro_gene_:5′UTR_gene_:gGene* fragments were PCR amplified from Col‐0 genomic DNA with the primers 1300‐pro‐in‐F and gGene‐3xFLAG‐in‐R, then inserted into the *Eco*RI/*Sac*I‐digested pCAMBIA1300‐35S‐3xFLAG vector through In‐Fusion cloning kit (ABclonal), to generate the intermediate construct pCAMBIA1300*‐Pro_Gene_:5′UTR_gene_:gGene‐3xFLAG*. Then, the corresponding *3′UTR_gene_* fragment was PCR amplified from Col‐0 genomic DNA with the primers FLAG‐3′UTR_gene_‐in‐F and FLAG‐3’UTR_gene_‐in‐R, and then inserted into the *Apa*I –digested pCAMBIA1300*‐Pro_gene_:5′UTR:‐gGene‐3xFLAG* intermediate vectors through In‐Fusion cloning, to obtain the pCAMBIA1300*‐Pro_gene_:5′UTR_gene_:gGene‐3xFLAG:3′UTR_gene_* constructs.

### Construction of the pCAMBIA1300‐Pro_PHR1_:5′UTR_mobile gene_:gPHR1‐3xFLAG:3′UTR_PHR1_ Vectors

The pCAMBIA1300*‐35S:gPHR1‐3xFLAG:3′UTR_PHR1_* intermediate vector was first constructed as follows: The *gPHR1‐3xFLAG:3′UTR_PHR1_* genomic fragment was amplified from the *pCAMBIA1300‐Pro_PHR1_:5′UTR_PHR1_:gPHR1‐3xFLAG:3′UTR_PHR1_* with the primers 1300‐in‐gPHR1‐F and FLAG‐3'UTR_PHR1_‐in‐R, then inserted into the *Eco*RI/*Apa*I‐digested pCAMBIA1300‐35Spro vector through In‐Fusion cloning (ABclonal).

Next, the *PHR1* promoter fragment was amplified from *pCAMBIA1300‐Pro_PHR1_:5′UTR_PHR1_:gPHR1‐3xFLAG:3′UTR_PHR1_* with the primers 1300‐in‐Pro_PHR1_‐EX‐F and 1300‐in‐Pro_PHR1_‐R. The 5′ UTR fragment from the genes producing mobile mRNAs was amplified from pCAMBIA1300*‐Pro_gene_:5′UTR:gGene‐3xFLAG:3′UTR_gene_* with the primers Pro_PHR1_‐in‐5′UTR‐F and Pro_PHR1_‐in‐5′UTR‐R. Finally, the *Pro_PHR1_* and *5′UTR_gene_* PCR amplicons were inserted into the *Apa*I‐digested pCAMBIA1300*‐Pro_PHR1_:gPHR1‐3′UTR_PHR1_* intermediate construct through In‐Fusion cloning (ABclonal) to obtain the pCAMBIA1300*‐Pro_PHR1_:5′UTR_mobile gene_:gPHR1‐3xFLAG:3′UTR_PHR1_* constructs.

### Construction of the pCAMBIA1300‐Pro_mobile gene_:5′UTR_PHR1_:gGene‐3xFLAG:3′UTR_mobile gene_ Vectors

First, the intermediate vector pCAMBIA1300*‐35S:gGene‐3xFLAG:3′UTR_mobile gene_* was constructed as follows: The *gGene‐3xFLAG:3′UTR_mobile gene_* fragment was amplified from *pCAMBIA1300‐Pro_mobile gene_:5′UTR_mobile gene_:gGene‐3xFLAG:3′UTR_mobile gene_* using primers 1300 in gene‐F and FLAG‐3'UTR_gene_‐in‐R. This fragment was then inserted into the *Eco*RI/*Apa*I‐digested pCAMBIA1300‐35Spro vector via In‐Fusion cloning (ABclonal).

Next, the gene promoter fragment was amplified from *pCAMBIA1300‐Pro_mobile gene_‐5’UTR_mobile gene_‐gGene‐3×FLAG‐3’UTR_mobile gene_* using primers 1300‐in‐Pro_gene_‐EX‐F and 1300‐in‐Pro_gene_‐R. The *PHR1 5′ UTR* fragment was amplified from pCAMBIA1300‐*Pro_PHR1_‐ 5’UTR_PHR1_‐gPHR1‐3×FLAG‐3’UTR_PHR1_* using primers gene pro in PHR1 5'UTR‐F and gene pro in PHR1 5'UTR‐R. Finally, the *Pro_mobile gene_* and *PHR1 5′UTR* PCR amplicons were inserted into the *Apa*I‐digested pCAMBIA1300*‐35S:gGene‐3xFLAG:3′UTR_mobile gene_* intermediate construct via In‐Fusion cloning (ABclonal) to generate the pCAMBIA1300*‐Pro_mobile gene_:5′UTR_PHR1_:gGene‐3xFLAG:3′UTR_mobile gene_* constructs.

All primers used for vector construction are listed in Table S19 (Supporting Information).

### Grafting Procedure and Phosphorus Limitation Treatments

Grafting experiments were performed as previously described,^[^ ^8^ ^]^ with some modifications. For homografting or heterografting, seedlings at 7 days after germination grown on plates as described above were cut transversely at the upper half of the hypocotyl, and scions (s) and rootstocks (r) were joined at this cut. Silicon tubing with a 0.5‐mm internal diameter was used to support the graft junction. Grafted seedlings were transferred onto new MS plates and placed into the growth chamber under the same temperature and light conditions described above for 11 days. Adventitious roots were regularly removed using surgical forceps. These grafted seedlings were then transferred to new MS plates for different extents of Pi starvation treatment: Pi‐sufficient (full phosphorus for 7 days [FP7D], serving as the negative control treatment, 1.25 mm Pi), 3 days of Pi deficiency (no phosphorus for 3 days [NP3D], 0 µm Pi), 7 days of Pi deficiency (no phosphorus for 7 days [NP7D], 0 µm Pi), and 7 days of Pi starvation (0 µm Pi) followed by 2 days of Pi (1.25 mm Pi) refeeding (NP7DRP2D).

### Accession Verification of Grafted Samples and Genomic Verification of Mutant Plants

Accession verification of grafted plants was determined by PCR‐based detection of SNPs based on published results.^[^ ^8^ ^]^ Briefly, the dCAPS products in AT2G01570 of grafted and ungrafted cDNA samples were amplified using gene‐specific primers: 5′–AGCGAGTATGCTTTTGTCTGTG–3′ and 5′–TTCGGTTTAGGTCTTGGTCCG–3′. The dCAPS RT‐PCR products were digested using *Rsa*I, resulting in 110‐bp and 135‐bp cDNA fragments for Col‐0 and a 244‐bp cDNA fragment for Ped‐0. Primers for genotyping of the *phr1* and *phl1* mutants were designed using the T‐DNA Primer Design software provided by the Salk Institute Genomic Analysis Laboratory (http://signal.salk.edu/tdnaprimers.2.html). The left (LP) and right (RP) genomic primers and the primer for identifying the T‐DNA insertion site are listed in Table s19 (Supporting Information).

### Ped‐0 Genomic DNA Sequencing, *de novo* Genome Construction, and Annotation

High‐quality genomic DNA was extracted from 7‐day‐old Ped‐0 plants using the cetyl trimethyl ammonium bromide (CTAB) method. Paired‐end libraries (150‐bp) were constructed and sequenced on an Illumina NovaSeq 6000 platform supplied by BIOZERON (http://www.biozeron.com/). For PacBio sequencing, genomic DNA was sheared into approximately 8‐ to 10‐kb fragments, and long‐read libraries were constructed using the SMRTbell Template Prep Kit (PacBio) and sequenced on the PacBio Sequel II platform.

Before genome reconstruction, the size, heterozygosity, and repetition rate of the genome was estimated based on the *K*‐mer distribution. Reads were trimmed using Trimmomatic v0.39 (https://github.com/usadellab/Trimmomatic/releases), and the next‐generation sequencing (NGS) short‐reads and Pacbio long‐reads were used for genome reconstruction with MaSuRCA v3.4.3 (http://www.genome.umd.edu/masurca.html). In addition, Pacbio long‐reads were assembled using Canu v2.1.1 (https://github.com/marbl/canu). The MaSuRCA and Canu results were then integrated using MUMmer v4.0 (http://mummer.sourceforge.net/). To increase the accuracy of the assembly, Illumina short reads were used to perform error correction using the default parameters of Pilon (v.1.22).^[^ ^65^ ^]^ The completeness of the genome assembly was then evaluated using BUSCOs v4.1.2 (https://busco.ezlab.org/)^[^ ^66^ ^]^ (Figure S1, Supporting Information).

Three programs, AUGUSTUS v3.2.3 (http://bioinf.uni‐greifswald.de/augustus/) (Stanke and Waack. 2003), Genewise v2.4.1 (https://www.ebi.ac.uk/seqdb/confluence/display/THD/GeneWise), and Trinity v2.11.0 (https://github.com/trinityrnaseq/trinityrnaseq/releases), were used for *de novo* gene predictions in the Ped‐0 genome. All predicted genes were integrated into a comprehensive nonredundant gene set using EVidenceModeler v1.1.1 (http://evidencemodeler.github.io/).^[^ ^67^ ^]^ Functional annotation of the protein‐coding genes in the Ped‐0 genome was performed using BLASTP (E value ≤ 1e^−5^) and the Swiss‐Prot (http://www.ebi.ac.uk/uniprot), NR (http://www.ncbi.nlm.nih.gov/), eggNOG (http://eggnogdb.embl.de/), and GO databases (http://geneontology.org/) databases.

### In Vitro Transcription of Anti‐rRNA Biotinylated Probes

The cDNA of rRNAs was amplified using listed primers containing the T7 promoter sequence (Table S20, Supporting Information). Next, these PCR products were gel purified and served as DNA templates for in vitro transcription. The reaction mixture (80 mm HEPES pH 7.5, 40 mm DTT, 25 mm MgCl_2_, 2 mm spermidine, 250 ng DNA templates, 4 mm ATP, 4 mm GTP, 4 mm CTP, 2 mm UTP, 2 mm biotin‐UTP [Lucigen], 1 U/µL SUPERase‐In RNase Inhibitor [ThermoFisher, Cat#AM2696], T7 RNA polymerase [NEB, Cat#M0251S]) was incubated at 37 °C overnight. After TURBO DNase (ThermoFisher, Cat#AM2238) treatment, the biotinylated RNA probes were purified with RNAClean XP beads (Bechman) and mixed. The final concentrations of anti‐rRNA probes are given in Table S20 (Supporting Information).

### RNA Extraction, Library Construction, and Sequencing

Total RNA of PCR‐verified grafted samples was extracted using TRIzol reagent (ThermoFisher) and pooled for each of three biological replicates per treatment (>20 grafted plants per biological replicate). Following treatment with TURBO DNase (ThermoFisher, lot. 91335471), 1 µg of DNase‐digested total RNA was subjected to ribosomal RNA (rRNA) depletion using homemade anti‐rRNA biotinylated probes. In brief, the mixture of total RNA and anti‐rRNA biotinylated probes was incubated in a thermocycler at 68 °C for 5 min, then ramped down by −0.1 °C/s to 22 °C, and finally held at 22 °C for 5 min. After this step, probe‐bound rRNAs were removed with streptavidin beads (ThermoFisher). Subsequently, the rRNA‐depleted RNAs were used to create RNA libraries using a Stranded mRNA‐seq Lib Prep Kit for Illumina (ABclonal, lot. NK2249L30W04). These libraries were then sequenced as 150‐bp paired‐end reads on a NovaSeq 6000 platform at Annoroad (https://www.annoroad.com/). For details of sample identification, read numbers, and mapping ratios, see Table S1 (Supporting Information).

### Accession‐Specific Construction of Reference SNPs and Identification of Long‐Distance Mobile RNAs

*Read processing*: Long‐distance mobile RNAs were defined as those traveling from scion to rootstock or vice versa. Paired‐end RNA‐seq data from homografts (rPed/sPed and rCol/sCol), heterografts (rCol/sPed, r*Col^RPL18^*/sPed, rCol/s*Ped^RPL18^*, and r*phr1 phl1*/sPed), and ungrafted samples (Col‐0 and Ped‐0) having experienced different Pi starvation treatments, as well as paired‐end genomic DNA‐seq datasets of Col‐0 and Ped‐0, were processed according to identical data workflows (Figure S1D, Supporting Information) and parameter settings. First, the raw reads were trimmed using Trim Galore v1.18 (http://www.bioinformatics.babraham.ac.uk/projects/trim_galore/) (parameters: ‐q 25 –phred33 –length 70 ‐e 0.1 –paired ‐o). Clean reads were then mapped to the genome reference (TAIR10_chr_all.fasta from TAIR) using the Burrows‐Wheeler aligner v0.7.17‐r1188 (http://bio‐bwa.sourceforge.net/) (parameters: bwa mem ‐M ‐t ‐R ‐o).^[^ ^68^ ^]^ SAMtools v1.11 (https://www.htslib.org/) was used with default parameters for sorting (samtools sort ‐@ ‐o) and indexing (samtools index ‐@).

*Variant calling and creation of accession‐specific reference SNPs*: For duplicate marking, the sorted and indexed. bam files from the RNA‐seq data (rPed/sPed, Col‐0, and Ped‐0) and the DNA‐seq data (Ped‐0 and Col‐0) were analyzed using the GATK v4.2.4.1 (https://gatk.broadinstitute.org/hc/en‐us) MarkDuplicates module. The alignment results were then used to call variants using the HaplotypeCaller module of GATK (parameters: ‐R –min‐base‐quality‐score 20 –native‐pair‐hmm‐threads 16 ‐I ‐O). The GATK SelectVariants module was used for SNP selection. InDels and chromosomal rearrangements were not considered. The VariantFiltration module in GATK was used for further SNP filtering (parameters: ‐R ‐V –filterexpression “QD < 2.0, MQ < 40.0, FS > 60., SOR > 3.0, DP < 10” ‐O). Unbiallelic SNP sites were removed using awk (Linux). SNPs occurring in both genome and transcriptome data were selected using bcftools isec (https://github.com/samtools/bcftools.git) and were considered accession‐specific reference SNPs for identification of mobile RNAs.

*Selection of candidate mobile RNAs and calculation of the mobility index*: To identify mobile RNAs, i.e., those transported between shoot and root, the paired‐end RNA‐seq reads of grafted plants (rCol/sPed, r*Col^RPL18^*/sPed, rCol/s*Ped^RPL18^*, r*phr1 phl1*/sPed) were analyzed based on the *Reads processing* section of the pipeline illustrated in Figure S1 (Supporting Information). SNPs were then called and filtered using GATK (parameters: “QD < 2.0, MQ < 40.0, FS > 60.0, SOR > 3.0”). The filtered SNPs were compared to the accession‐specific reference SNPs to identify the corresponding SNPs in the mobile RNAs. To accurately select the SNPs, the Baymobil^[^ ^45^, ^46^ ^]^ (github.com/mtomtom/baymobil) tool was used to exclude PCR or sequencing errors by comparing sequencing data from homograft and hetrograft samples. The SNPs with a log_10_Bayes factor > 1 were selected and considered informative SNPs for investigation. Reads covered by informative SNPs were identified using the SAMtools view module accompanied by SAMtools calmd and grep (parameters: samtools view ‐b | samtools calmd ‐e ‐ $genome_reference.fa ‐b |samtools view ‐| grep ‐v “^@”|awk ‐v pos=“$SNP_position” ′BEGIN {OFS = FS = “∖t”}; {n=split($10,a,″″); if(a[(pos‐$4)+1] != “=”) print pos,(pos‐$4)+1, a[(pos‐$4)+1], $1, $4, $10}′ >> reads‐info.txt). SNP‐containing reads were extracted using seqtk subseq (https://github.com/lh3/seqtk.git) and mapped to the reconstructed genome (Ped‐0) or the TAIR10 genome for identification of mobile RNAs. The coverage of reads for mobile RNAs was calculated using featureCounts v2.0.1 (https://subread.sourceforge.net/featureCounts.html). Mobile transcripts that were covered by more than three SNP‐containing reads and detected in at least two biological replicates were considered high‐confidence mobile transcripts. The abundance of a mobile RNA (mobile abundance) was calculated by mapping the reads covering the high‐confidence SNPs to the reference genome corresponding to the donor tissue. The mobile index (MI) was defined as the mean mobile abundance in recipient tissue/mean local abundance in donor tissue.

*Identification of mobile transcripts using dCAPS markers*: dCAPS maker sites were identified by comparing the coding sequences (CDSs) of Col‐0 (TAIR10) and Ped‐0. To identify PSR‐specific mobile RNAs, the 241‐bp RT‐PCR products for *PORB* (AT4G27440) and 263‐bp products for *LHCB5* (AT4G10340) were amplified from total RNA extracted from the roots of Col‐0, Ped‐0, and heterografted plants and digested with *Bam*HI. Digestion of the *PORB* RT‐PCR products from Col‐0 produced fragments of 114 and 127 bp, and digestion of the *LHCB5* RT‐PCR products of Ped‐0 produced fragments of 137 and 126 bp.

*Analysis of differential gene expression*: The cleaned RNA‐seq reads were mapped to the TAIR10 genomes and homemade Ped‐0 genome using Hisat2 v2.1.0 (https://daehwankimlab.github.io/hisat2/). After sorting and indexing using SAMtools v1.11, read coverage was calculated using featureCounts v2.0.1 and then normalized using DEseq2 (https://bioconductor.org/packages/release/bioc/html/DESeq2.html). Differentially expressed genes were identified with a cutoff of |log_2_fold‐change| > 1 and a *P*‐value < 0.05.

### Immunoblotting

Immunoblotting was performed as described previously.^[^ ^69^ ^]^ The primary antibody for immunoblotting was mouse anti‐FLAG (ABclonal, Cat#AE005), and the secondary antibody was goat anti‐mouse IgG (ThermoFisher, Cat#31430).

### Translating Ribosome Affinity Purification and Sequencing (TRAP‐seq)

To identify the specific mRNAs being translated, transgenic plants harboring a transgene encoding 3x FLAG‐tagged ribosomal protein RPL18 driven by the cauliflower mosaic virus (CaMV) 35S promoter were created in both the Col‐0 and Ped‐0 backgrounds (*35S_pro_:3xFLAG‐RPL18*/Ped, named *Ped^RPL18^*, and *35S_pro_:3xFLAG‐RPL18*/Col, named *Col^RPL18^*). Hypotocol grafting to produce chimeric plants (rCol/s*Ped^RPL18^* and r*Col^RPL18^*/sPed) and Pi starvation treatments were performed as described above. Shoot and root samples were collected and placed into liquid nitrogen and stored at −80 °C. TRAP‐seq was performed according to a published method^[^ ^51^ ^]^ with some modifications. Briefly, 2 g of powdered sample was suspended in 2 mL IP buffer (200 mm Tris‐HCl pH 9.0, 200 mm KCl, 35 mm MgCl_2_, 25 mm Na_2_EGTA, 1 mm DTT, 100 µg mL^−1^ cycloheximide [J&K Scientific], 1 pellet of protease inhibitor [Roche, 713260] per 10 mL, 1% [v/v] Triton X‐100) on ice, and samples were protected from light. The suspended samples were placed into cold 1.5‐mL tubes, incubated on ice for 10 min, and centrifuged twice at 12 000 rpm for 15 min at 4 °C. The supernatants were transferred into new 1.5‐mL tubes, after which 5 µL mL^−1^ TURBO DNase (ThermoFisher, Cat#AM2238) and 1/100 (v/v) SUPER‐In RNase inhibitor (ThermoFisher, Cat#2696) were added and incubated at room temperature for 30 min. At the same time, anti‐FLAG‐conjugated magnetic beads (Sigma, lot. SLLF7243, 180 µL in total) were equilibrated three times in RNID W/O PI buffer (20 mm Tris‐HCl pH 8.0, 140 mm KCl, 35 mm MgCl_2_, 50 µg mL^−1^ cycloheximide, 50 µg mL^−1^ Chloramphenicol [Chl]). The equilibrated anti‐FLAG‐conjugated magnetic beads were added to the digested samples and incubated at 4 °C for 2 h with gentle rotation. The beads, now containing ribosome–RNA complexes, were washed three times with 1 mL wash buffer (20 mm Tris‐HCl pH 8.0, 140 mm KCl, 35 mm MgCl_2_, 50 µg mL^−1^ cycloheximide, 50 µg mL^−1^ Chl, 0.5 pellet/20 mL EDTA‐free proteinase inhibitor cocktail [Roche]) at 4 °C for 5 min. Ribosome‐associated RNA was extracted using TRIzol reagent (ThermoFisher), and TRAP‐seq libraries were constructed according to the RNA‐seq procedure described above and sequenced in paired‐end format as 150‐bp reads on a NovaSeq 6000 platform at Annoroad (https://www.annoroad.com/). Mobile and translatable transcripts were identified using a custom pipeline (Figure S1, Supporting Information). Translational efficiency (TE) was calculated based on the abundance in TRAP‐seq and RNA‐seq data.

### RNA Secondary Structure, GC Content, and Length Analysis

The coding sequences (CDS), 5′ UTR and 3′ UTR of transcripts were extracted using the Fasta Extract function in TBtools v2.012^[^ ^70^ ^]^ based on the *Arabidopsis* TAIR10 5′ UTR (TAIR10_5_utr_20101028.txt), coding sequence (TAIR10_cds_20101214_updated.txt), and 3′ UTR (TAIR10_3_utr_20101028.txt) reference sequences in TAIR (https://www.arabidopsis.org/index.jsp).

To calculate minimum thermodynamic free energies, RNA sequences were folded using the RNAfold function of the ViennaRNA package, as previously reported.^[^ ^53^ ^]^ ΔG was defined as the minimum free energy of the RNA secondary structure as determined by the default RNAfold function within the ViennaRNA package 2.6.3.^[^ ^53^ ^]^ Minimum free energy values were divided by sequence lengths to calculate –ΔG/nt values. The GC contents were calculated using the Bio.SeqUtils function in Biopython (https://biopython.org/). Lengths were extracted using the vlookup function in Excel. Modeled RNA secondary structures of selected 5′ UTRs were drawn using VARNA (http://varna.lri.fr/) or RNArtist (https://github.com/fjossinet/RNArtist).

### Target‐Specific DMS‐MaPseq

Target specific DMS‐MaPseq was performed as described.^[^ ^53^, ^54^ ^]^ Root and shoot samples from 21‐day‐old WT or *phr1phl1* plants were harvested and treated with 1% DMS (MACKLIN) or water as a control for 20 min under vacuum (≈12 psi). DMS reacts with unpaired adenosines (A) and cytosines (C) in RNA in vivo. To stop the DMS treatment, β‐Mercaptoethanol (Sigma) was added to the reaction mixtures to a final concentration of 20%, followed by incubation under vacuum for 5 min. After washing three times with DEPC‐treated water, samples were frozen in liquid nitrogen. Two biological replicates were prepared for each DMS‐treated sample, and one biological replicate was prepared for each no DMS‐treated sample.

DMS‐treated and untreated samples were ground into powder. Total RNA was purified using Trizol reagent (Thermo). After DNase treatment, ≈10 µg of RNA was mixed with 0.5 µl of 10 µm gene‐specific RT primers mixture. The mixture was precipitated and resuspended in 10 µl of Tris‐HCl solution (50 mm KCl, 10 mm Tris‐HCl, pH 7.5). The solution was heated at 75 °C for 3 min, followed by incubation at 57 °C for 15 min. Then, 4 µl of 5xFirst‐Strand buffer (Thermo), 1 µl of 0.1 m DTT, 1 µl of SUPERase‐In RNase inhibitor (Thermo), 1µl of RNase‐free H_2_O, and 1 µl of TGIRT‐III (InGex) were added. After incubation at room temperature for 30 min, 2 µl of 10 mm dNTPs were added, and reverse transcription was conducted at 60 °C for 2.5 h. The reaction was stopped by adding 2 µl of 2.5 m NaOH to degrade the RNA, followed by neutralization with 5 m HCl. The cDNA was purified using RNAClean XP beads and used for target specific PCR and DNA libraries construction.

The 5’UTR of PHR‐mediated PSR‐specific and non‐PHR‐mediated mobile mRNA, as well as 18S rRNA, were amplified using KOD FX DNA polymerase (TOYOBO) with target‐specific primers. PCR bands were gel‐purified and normalized according to band intensity before library construction. Equal amounts of PCR products were mixed, and DNA libraries were constructed using the Rapid Plus DNA Lib Prep Kit for illumine V2 (ABclonal). Target‐specific DMS‐MaPseq libraries were sequencing in 2 x 150‐nt paired‐end mode on the NovaSeq platform at Annoroad.

All primers used for target‐specific RT and target‐specific PCR are listed in Table S19 (Supporting Information).

### Target‐Specific DMS‐MaPseq Data Analysis

The target‐specific DMS‐MaPseq data was analyzed as described with some modifications.^[^ ^54^, ^71^ ^]^ Briefly, raw reads were filtered using TrimGalore (v0.6.6), and clean reads were mapped to the corresponding target sequences using TopHat (v2.1.1) with allowing for 10% mismatches. Next, uniquely mapped reads were extracted from the BAM file using the Linux command grep with NH1:1 tag. Mutations and sequencing depth were counted from the uniquely mapped BAM file after discarding mismatches located within 3nt of an indel. DMS reactivity for each A and C nucleotide was calculated as the ratio of mismatch to sequencing depth.

Target‐specific DMS‐MaPseq was performed to profile the 5’UTR RSS of five PHR‐mediated PSR‐specific mobile mRNAs (AT1G55670, AT3G14420, AT4G10340, AT4G27440, AT5G01530) and five non‐PHR‐mediated mobile mRNAs (AT1G60170, AT2G07727, AT2G35750, AT3G41762, AT3G51780). Due to PCR bias caused by low expression, some replicates showing abnormal DMS reactivity‐such as those where the mutation rates of a few A and C were dramatically high while whose of other As and Cs were extremely low (<0.5%)‐were excluded. Reliable DMS‐MaPseq data (32 datasets in total) were ultimately obtained for three PHR‐mediated PSR‐specific mobile mRNAs (AT1G55670, AT3G14420, AT5G01530) and five non‐PHR‐mediated mobile mRNAs (AT1G60170, AT2G07727, AT2G35750, AT3G41762, AT3G51780) in WT and *phr1 phl1* under FP and NP conditions (Figures S12 and S13, Supporting Information). For RSS data with two DMS+ biological replicates, the DMS reactivity for each A and C was averaged. Normalization was then performed to obtain normalized DMS reactivity for all 32 DMS‐MaPseq datasets. For each dataset, all raw DMS reactivities were divided by the median of the highest 5% of mutation rates to compute the normalized DMS reactivities. Normalized reactivities >1.0 were winsorized by setting them to 1.0. Based on the normalized DMS reactivities, the RSS were modelled using RNAStructure (v6.5).^[^ ^55^ ^]^ Normalized DMS reactivities were color‐coded on structure models using VARNA (http://varna.lri.fr,).

### Statistical Analysis

Statistical analysis was performed using Excel (Microsoft) or GraphPad Prism 10. The statistical details, including the statistical tests used, the exact value of *n* for each measurement, and the *P*‐values, are presented in the figure legends and figures. Statistically significant differences are indicated as * *P* < 0.05; ** *P* < 0.01; *** *P* < 0.001; ns, not significant. All data are presented as the means ± SD.

## Conflict of Interest

The authors declare no conflict of interest.

## Author Contributions

W.D. and S.W. contributed equally to this work. Z.W. conceived the project and designed the experiments; W.D. performed the major of the experiments and conducted the bioinformatics analysis, with S.W., L.Q., K.L. and Q.J.; S.W. and W.D. performed the target‐specific DMS‐MaPseq and data analysis; S.W. performed the vector construction and development of transgenic lines; C.L. and X.S. assisted in the bioinformatics analysis for the identification of mobile RNAs; K.Y. and X.M. provided intellectual support; W.D., Z.W. and S.W. wrote the manuscript.

## Supporting information



Supporting Information

Supporting Information

Supporting Information

Supporting Information

Supporting Information

## Data Availability

All data are available in the main text or the supplementary materials. The raw sequencing data generated in this study had been deposited in the Genome Sequence Archive (GSA) under accession code CRA021227. Genetic materials will be available when requested.
