# Supplementary material for: PHR‐Mediated Pi Starvation Response Mobile Messenger RNAs Represent Noncoding Transcripts in Recipient Tissues
Source: Adv Sci (Weinh). 2025 Nov 30;13(4):e02911. doi: 10.1002/advs.202502911 (PMC12822437; doi:10.1002/advs.202502911)
Supplement: Supplementary file 1 — Supporting Information [file ADVS-13-e02911-s002.pdf]

Supporting Information for

PHR-mediated Pi starvation response mobile messenger RNAs represent noncoding transcripts in recipient tissues

Weiguo Dong[1,†], Shuman Wang[1,†], Chao Liu[2], Lulu Qiao[1], Kai Liu[1], Qiongli Jin[1], Xiaorong Mo[1], Xingxing Shen[2], Keke Yi[3], Zhiye Wang[1,*]

[1]State Key Laboratory of Plant Environmental Resilience, College of Life Sciences, Zhejiang University, Hangzhou, Zhejiang, 310058, China

[2]Key Laboratory of Biology of Crop Pathogens and Insects of Zhejiang Province, College of Agriculture and Biotechnology, Zhejiang University, Hangzhou, Zhejiang, 310058, China

[3]State Key Laboratory of Efficient Utilization of Arid and Semi-arid Arable Land in Northern China/Key Laboratory of Plant Nutrition and Fertilizer, Ministry of Agriculture, Institute of Agricultural Resources and Regional Planning, Chinese Academy of Agricultural Sciences, Beijing, 100097, China

[†] These authors contributed equally: Weiguo Dong, Shuman Wang

[*] E-mail: wangzhiye1@zju.edu.cn (Z.W.)

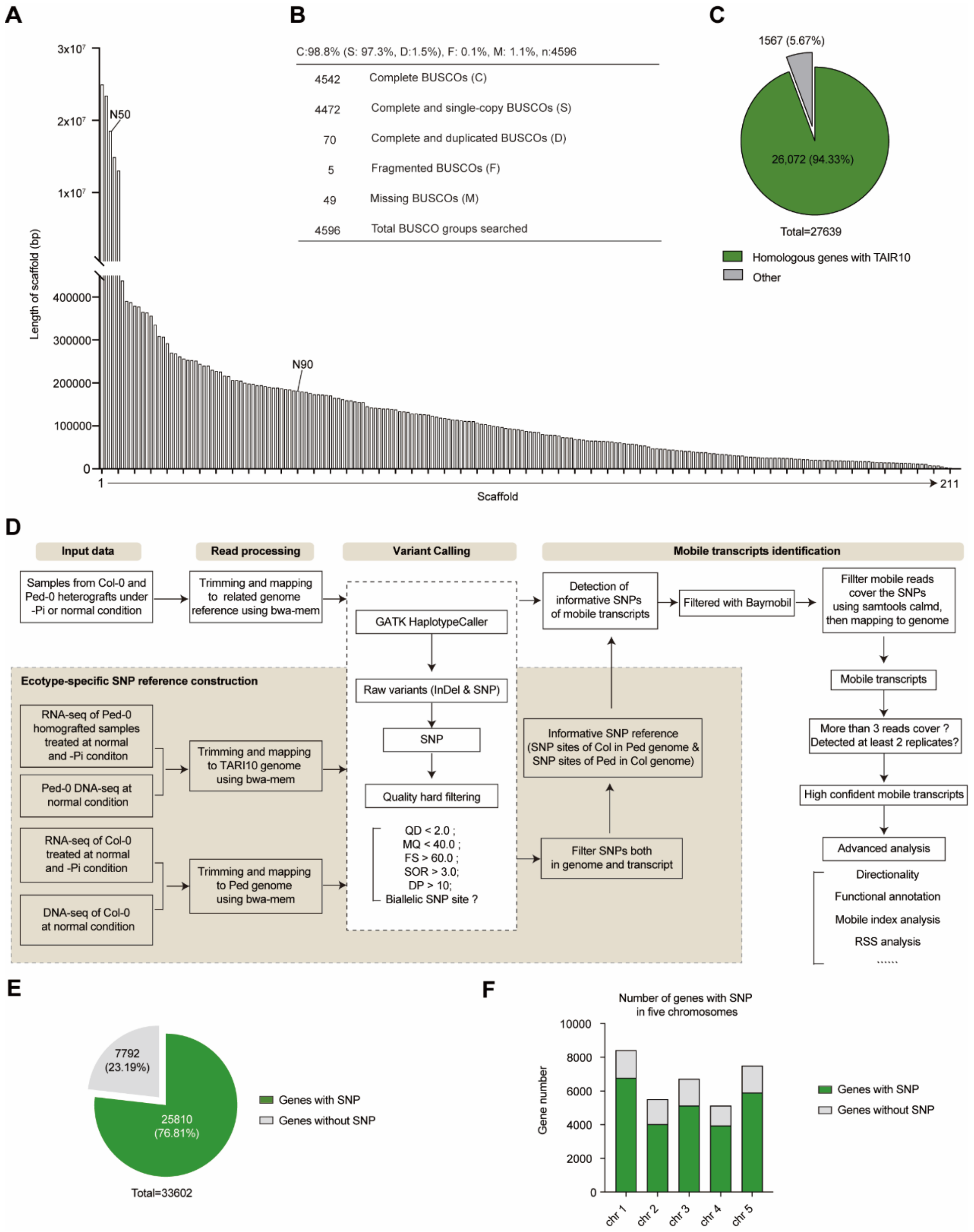

Figure S1. Construction of an accession-specific SNP reference list between Ped-0 and Col-0.

A, Distribution of scaffold length for the constructed Ped-0 genome. The five longest genomic scaffolds are of similar length as the five *Arabidopsis* chromosomes from Col-0 (TAIR10). N50 or N90 refers to the length of the shortest scaffold of all scaffolds that is at least half (N50) or 90% (N90) of the lengths of all scaffolds. B, Summary of BUSCO score analysis of single-copy orthologous genes in the Ped-0 genome,

indicating its high genome integrity. C, Pie chart showing the number of homologous genes in Ped-0 compared to TAIR10. D, Diagram of the custom bioinformatic pipeline used for transcriptome-wide identification of mobile RNAs. E, Pie chart showing that 76.8% of TAIR10-annotated genes (25,810/33,602) have informative SNPs between Col-0 and Ped-0 (green). F, SNP analysis showing a similar distribution of genes with informative SNPs across the five *Arabidopsis* chromosomes.

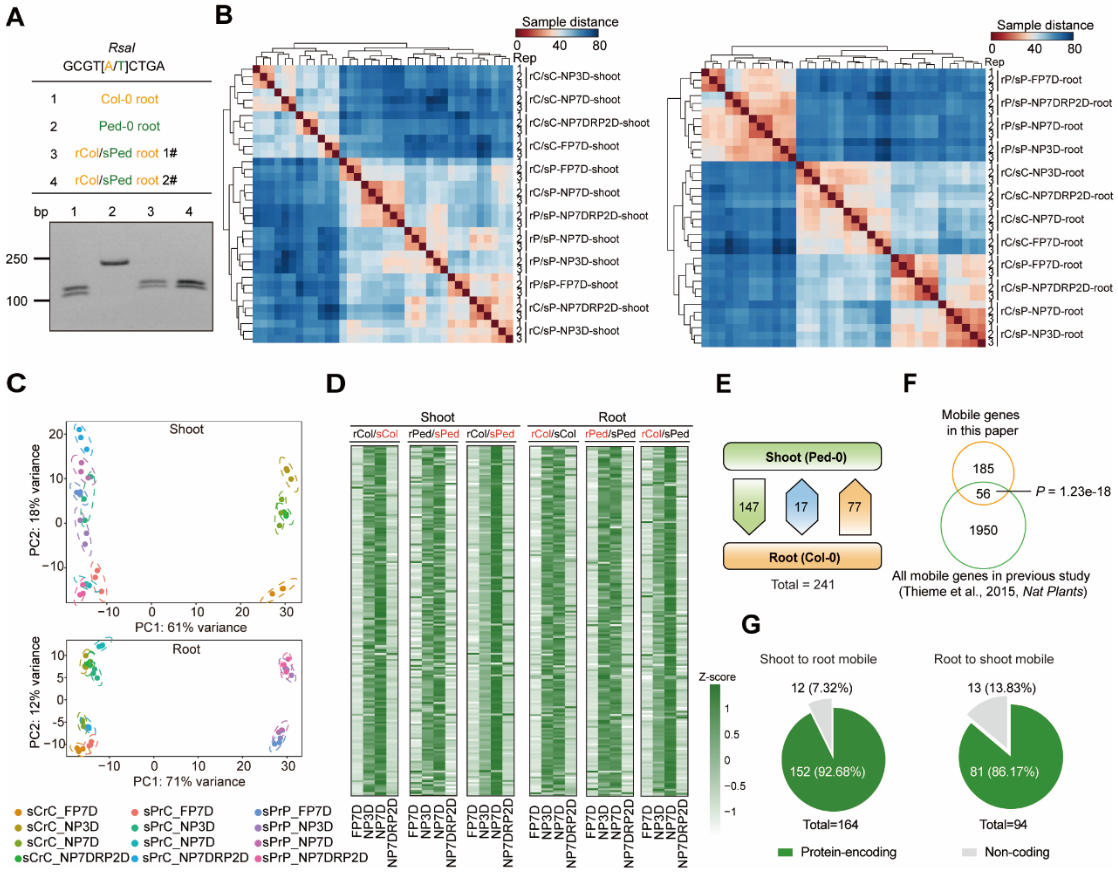

Figure S2. Transcriptome-wide identification of long-distance mobile RNAs in *Arabidopsis*.

A, Genotype verification of harvested heterograft materials using a CAPS marker. The bands indicate the PCR products of Col-0, Ped-0 and rCol/sPed, respectively, after digestion by *Rsa*I. B and C, Clustering (B) and principal component analysis (PCA) (C) showing high reproducibility among the three biological replicates of homograft and heterograft samples. D, Relative PSR marker gene expression based on RNA-seq in shoot (left) and roots (right) in Col and Ped homografts and heterografts. E, Number of transcripts moving bidirectionally (blue) or unidirectionally into root (green) or shoot (orange) tissues. F, Venn diagrams showing the significant overlap of identified mobile RNAs between this study (n = 241) and 2015 Nat. Plants published data[8] (n = 2006). *P*-values were calculated using the hypergeometric test. G, Pie charts showing that most shoot-to-root mobile RNAs (92.7%) and most root-to-shoot mobile RNAs (86.2%) are protein-coding RNAs. rC/sP, rCol/sPed; rP/sP, rPed/sPed; rC/sC, rCol/sCol.

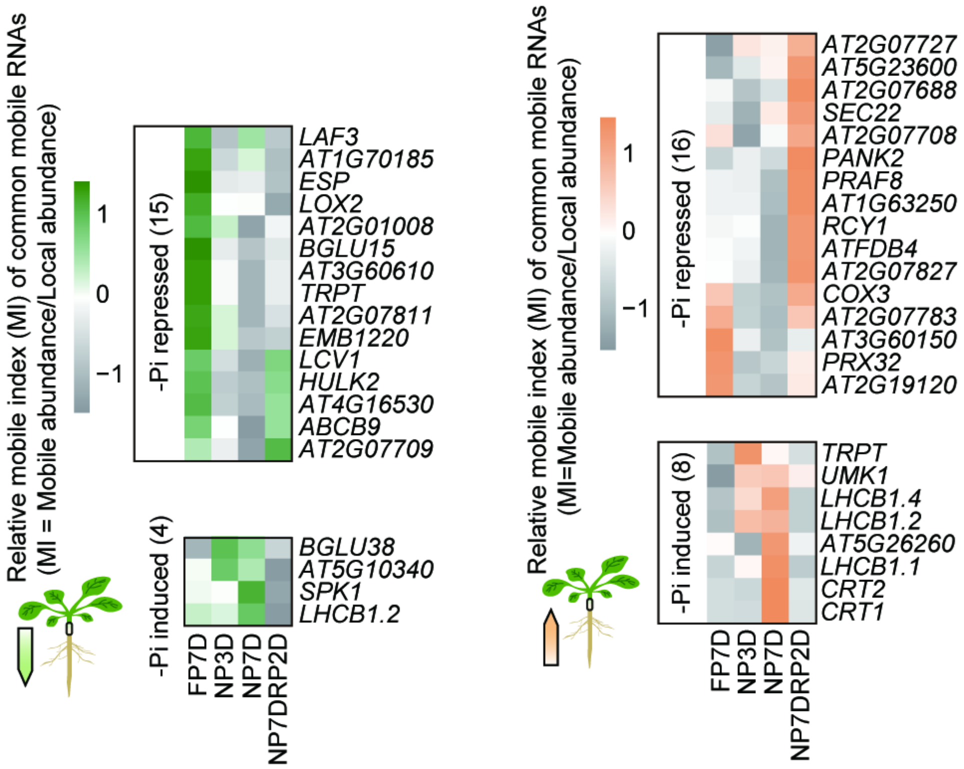

Figure S3. Mobile index (MI) of the common mobile transcripts.

Mobile indices of shoot-to-root and root-to-shoot common mobile RNAs. MI = mobile RNA abundance/local abundance in donor tissue.

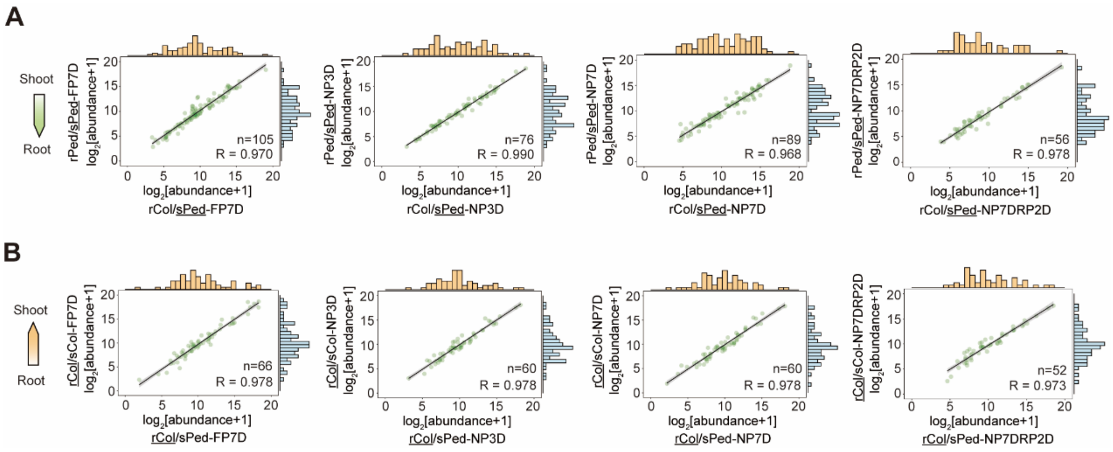

Figure S4. Heterografting does not change the local expression levels of mobile RNAs in the donor tissue.

A, Scatterplots showing the correlation between the local transcript abundance of shoot-to-root mobile transcripts in rPed/sPed shoot and rCol/sPed shoot samples. B, Scatterplots showing the correlation between the local expression of root-to-shoot mobile transcripts in rCol/sCol root and rCol/sPed root samples. *R* values listed in the plots are the Pearson's correlation coefficients for the linear regression. *n* values listed in plots indicate the number of mobile transcripts in different conditions.

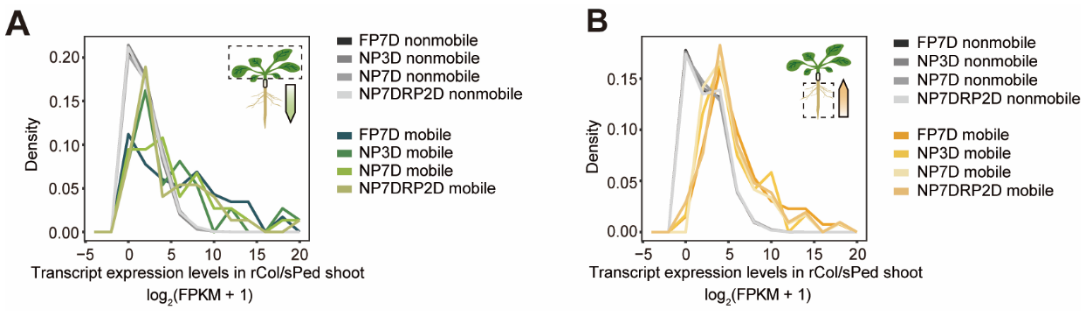

Figure S5. The mobile RNAs correspond to highly expressed genes in donor tissues. A and B, Abundance levels of mobile RNAs and nonmobile RNAs, estimated as FPKM (fragments per kilobase transcript per million mapped reads) from RNA-seq data suggesting that mobile RNAs are relatively highly expressed in shoots (A) and roots (B).

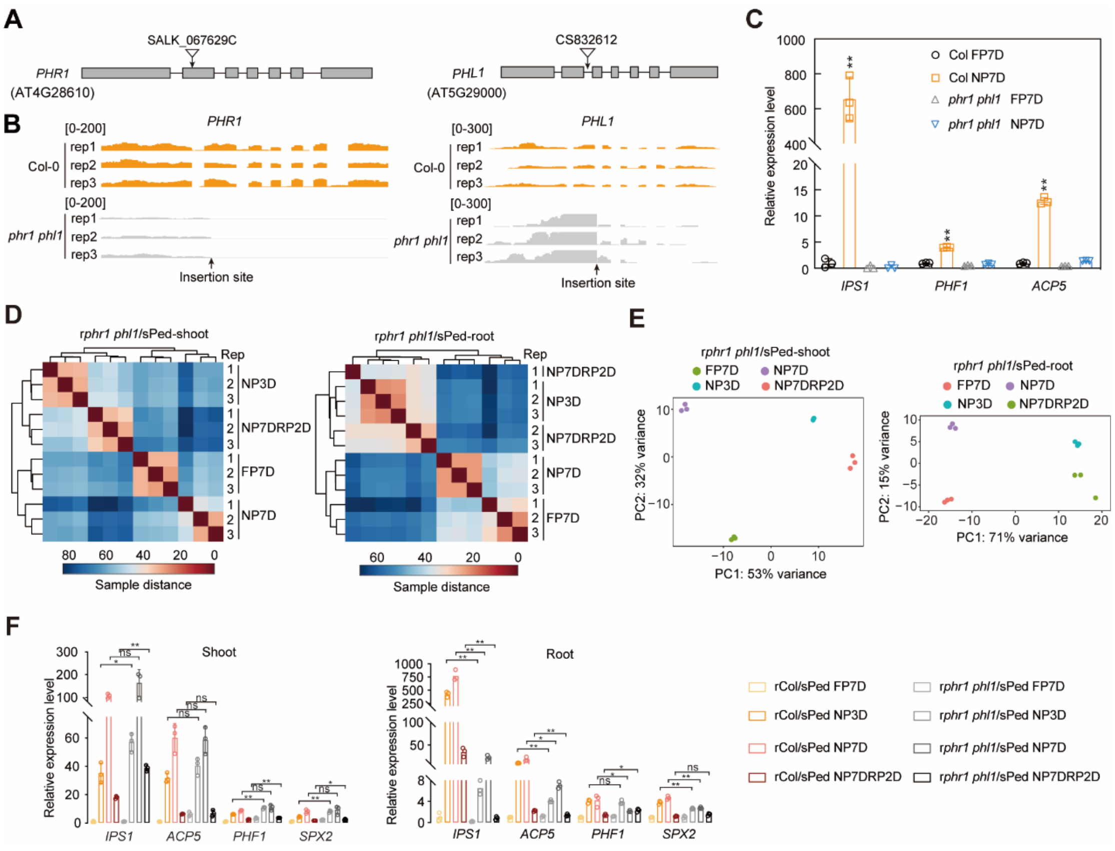

Figure S6. Identification of the *phr1 phl1* mutant and validation of the high-quality RNA-seq data from r*phr1 phl1*/sPed samples.

A, Diagrams showing the T-DNA insertion sites in the *phr1* and *phl1* mutants. B, Integrative Genomic Viewer windows showing the read coverage along *PHR1* (left) and *PHL1* (right) in Col-0 and *phr1 phl1*. C, Relative expression of PSI genes in Col-0 and *phr1 phl1* under Pi-sufficient and Pi-deficient conditions. The relative expression of PSI genes was calculated with SD from three biological replicates. *, $P < 0.05$, **, $P < 0.01$, two-tailed Student's *t*-test. D and E, Clustering (D) and PCA (E) showing high reproducibility among the three biological replicates for r*phr1 phl1*/sPed samples. F, Relative expression of selected PSI genes in shoots (left) and roots (right) of r*phr1 phl1*/sPed and rCol/sPed samples under Pi deficiency. Values are means ± SD from three biological replicates. *, $P < 0.05$, **, $P < 0.01$, two-tailed Student's *t*-test.

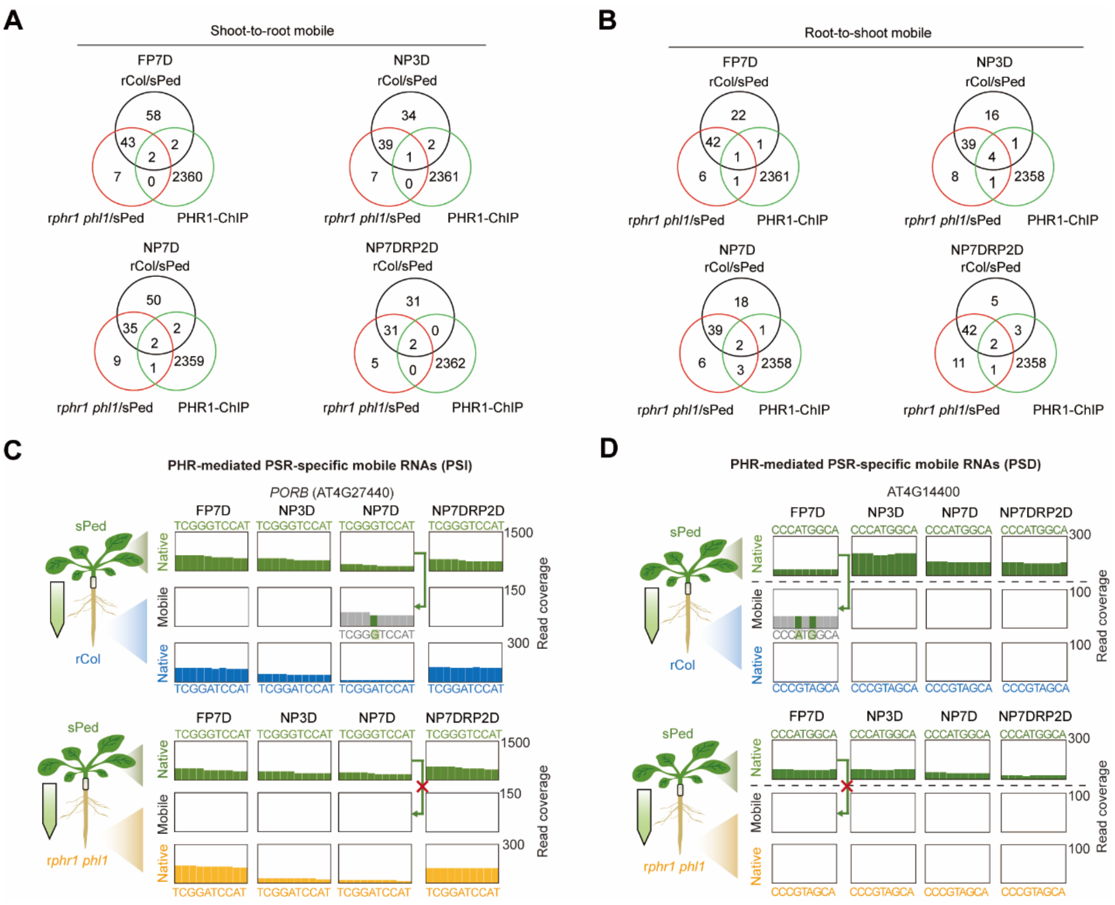

Figure S7. PHR-mediated mobile RNAs are not produced from PHR1 direct target loci, and selected examples of PHR-mediated PSR-specific mobile RNAs.

A and B, Venn diagrams showing the overlap of identified shoot-to-root (A) and root-to-shoot (B) mobile RNAs among rCol/sPed, r*phr1 phl1*/sPed, and PHR1-ChIP data. Most PHR-dependent mobile RNAs are direct targets of PHR1. C and D, IGV browser windows of read coverage for informative SNPs in the coding sequence of a PHR-mediated PSI mobile mRNA (*PORB*) (C) or a PSD mobile mRNA (AT4G14400) (D). Each plot represents the cumulative read count from Col-0 (blue bars), Ped-0 (green bars), or *phr1 phl1* (orange bars). In mobile boxes, gray bars indicate identical nucleotides between Ped-0 and Col-0, and green bars indicate the informative SNPs between Ped-0 and Col-0. Directions of green arrows show the predicted transcript movement from shoot (sPed) to root (rCol or r*phr1 phl1*).

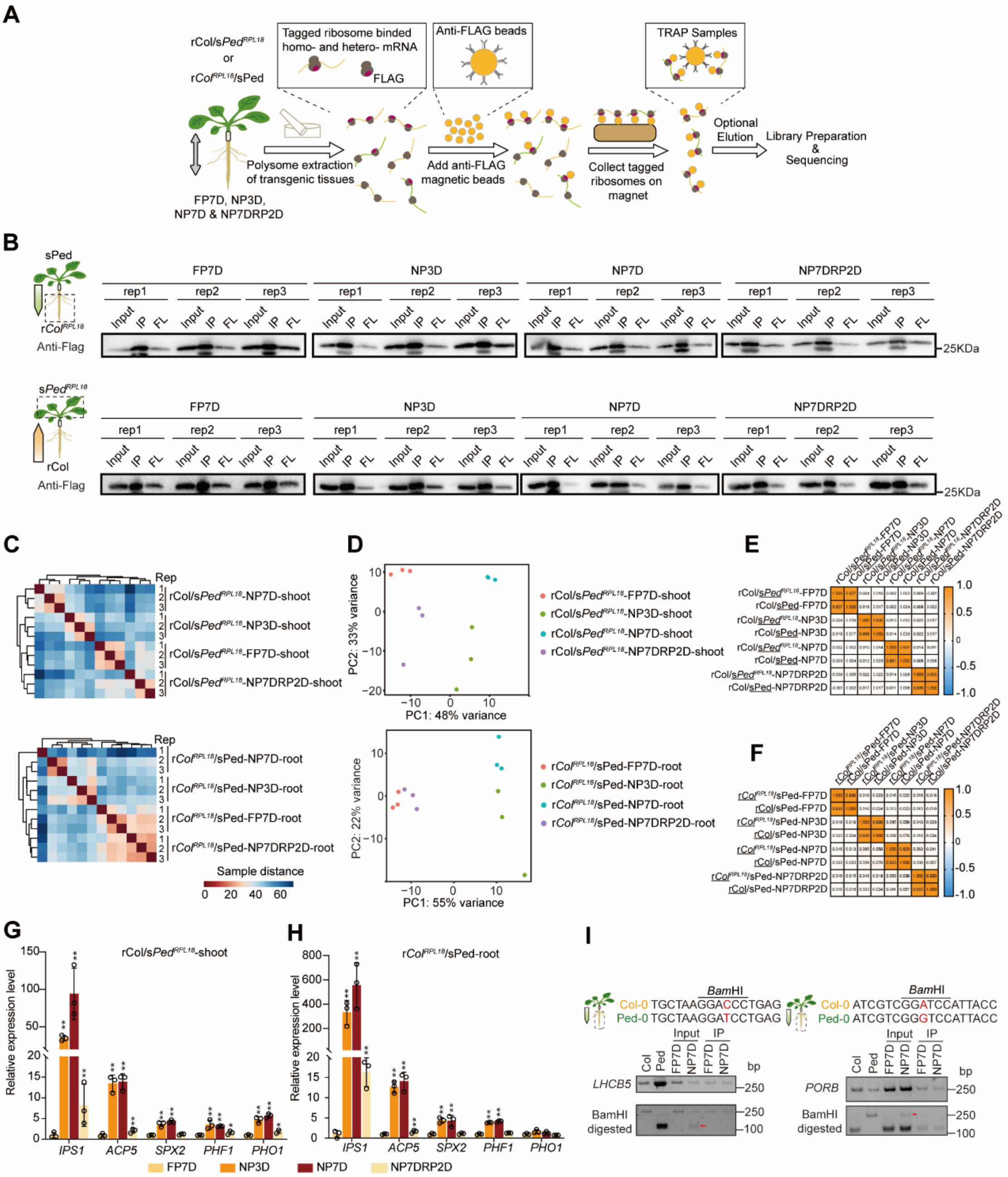

Figure S8. High quality of TRAP-seq data from heterograft samples.

A, Diagram indicating the experimental strategy for TRAP-seq combined with heterografting between non-transgenic wild-type and *35S:3×FLAG-RPL18* transgenic plants. B, Immunoblots showing highly efficient affinity purification of polysome–RNA complexes. IP, immunoprecipitate; FL, flow-through. C and D, Clustering (C) and PCA (D) showing high reproducibility among the three biological replicates of TRAP-seq samples. E and F, High correlation between TRAP-seq data and related RNA-seq data in shoot (E) and root (F) samples. G and H, Relative abundance of PSR transcripts

bound to ribosomes in rCol/s*Ped*^{RPL18} shoot (G) and r*Col*^{RPL18}/sPed root (H) samples during Pi sufficiency and Pi deficiency. Values are means ± SD from three biological replicates. *, $P < 0.05$, **, $P < 0.01$, two-tailed Student's *t*-test. I, Identification of mobile transcripts in TRAP-input and TRAP-IP samples based on CAPS marker analysis. The red arrows indicate the band for the mobile transcript. The SNP for the CAPS marker is highlighted in red. In B and C, rep means biological replicate.

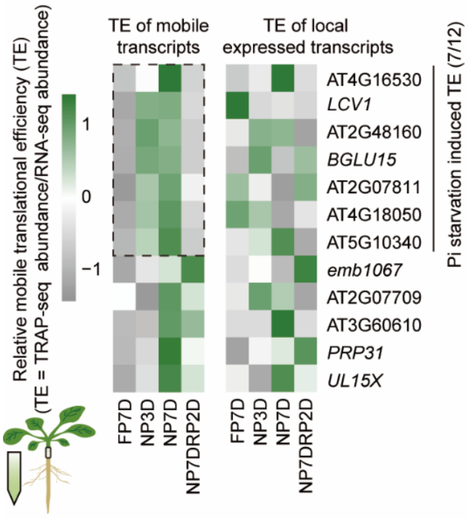

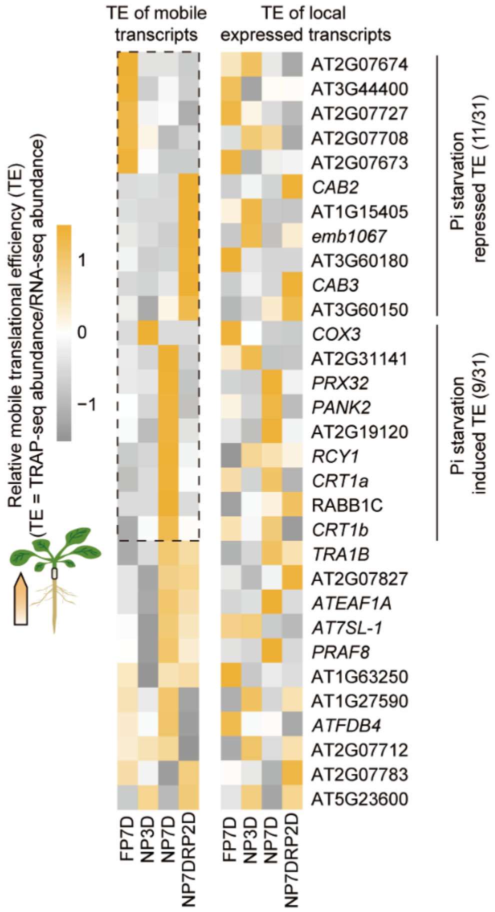

Figure S9. Translational efficiency of common mobile transcripts and their corresponding locally expressed transcripts.

Heatmaps showing the translational efficiency (TE) of shoot-to-root or root-to-shoot translated mobile RNAs and that of their corresponding locally expressed RNAs in recipient tissues under Pi deficiency. TE, RNA abundance in TRAP-seq/RNA abundance in RNA-seq.

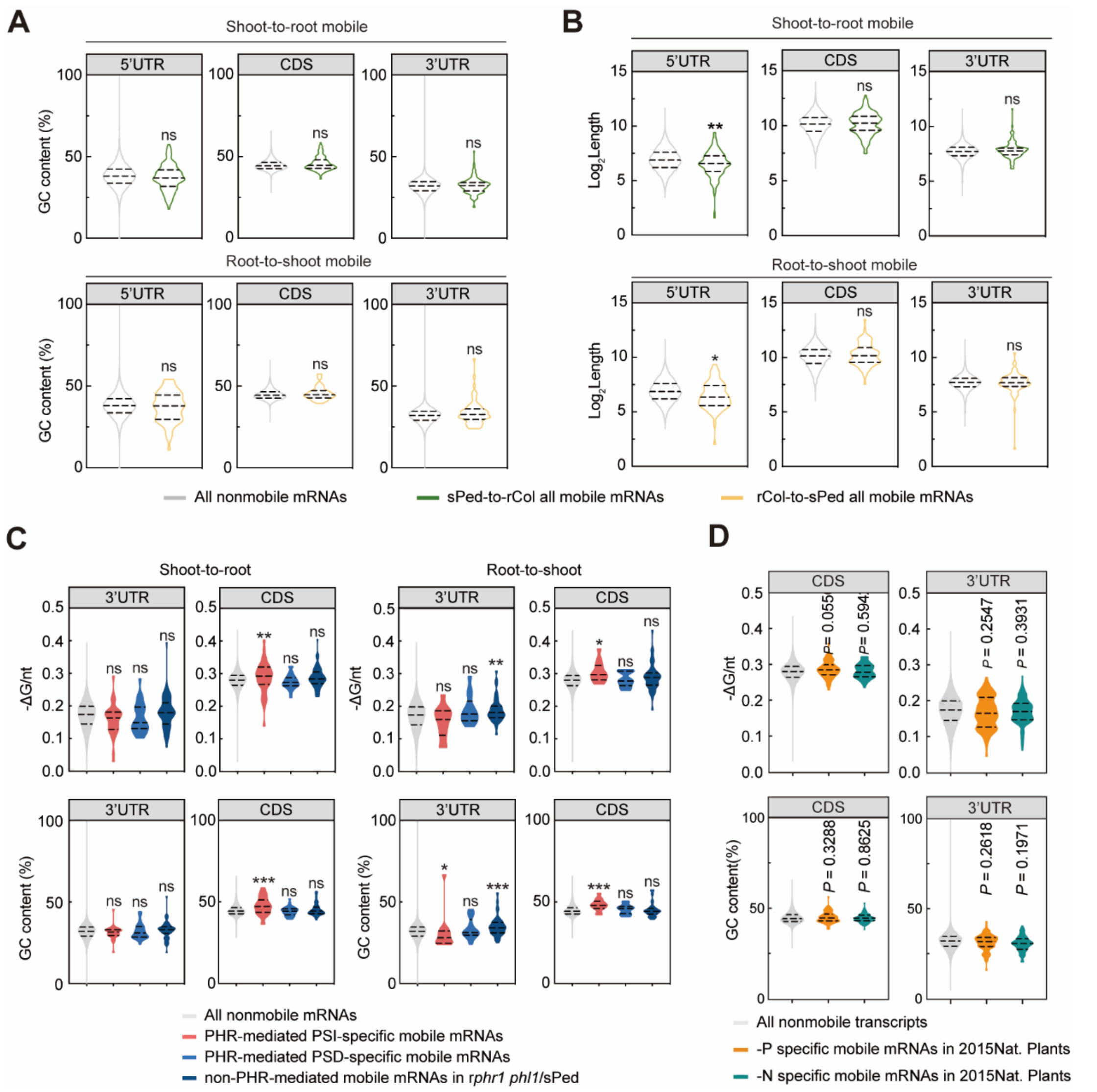

Figure S10. RSS, GC content, and length analysis of mobile and nonmobile RNAs. A, Violin plots showing the lack of difference in GC content for the 5′ UTR, CDS, and 3′ UTR between mobile and nonmobile RNAs. B, Violin plots showing a slight but significant difference for 5′ UTR length between mobile and nonmobile RNAs. C, Differences in −ΔG/nt (upper panel) and the GC content (bottom panel) of the CDS and 3′ UTR among PHR-mediated PSR-specific mobile, non-PHR-mediated mobile, and nonmobile RNAs. D, Absence of difference in −ΔG/nt (upper panel) and GC content (bottom panel) for the CDS and 3′ UTR among published −P-specific mobile, −N-specific mobile, and nonmobile RNAs in the 2015Nat. Plants study[8]. *P*-values were calculated using the Wilcox test. *, $P < 0.05$, **, $P < 0.01$, ***, $P < 0.001$, ns, not significant. All data for analysis are provided in Supplementary Tables 13-18.

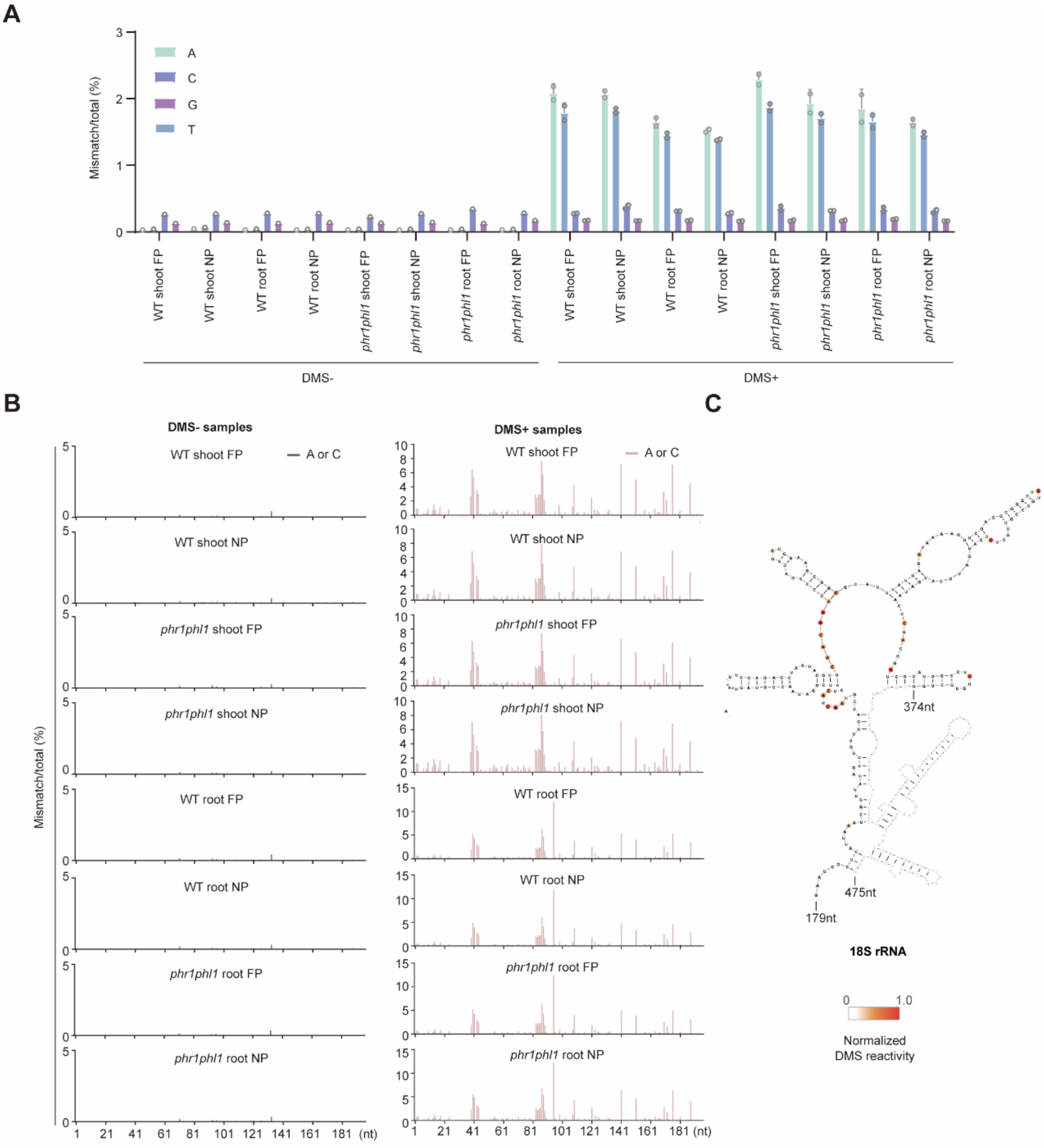

D

	Mutation/Total															
	WT shoot FP		WT shoot NP		*phr1phl1* shoot FP		*phr1phl1* shoot NP		WT root FP		WT root NP		*phr1phl1* root FP		*phr1phl1* root NP	
	High	Low	High	Low	High	Low	High	Low	High	Low	High	Low	High	Low	High	Low
Single-stranded	79.49% (86.11%)	30.43%	80% (87.5%)	15.79%	83.87% (90.32%)	26.67%	83.33% (90%)	30%	78.57% (90.48%)	16.67%	72.22% (85.19%)	25%	79.41% (88.24%)	26.92%	81.08% (89.19%)	20%
Base-paired	20.51% (13.89%)	69.57%	20% (12.5%)	84.21%	16.12% (9.68%)	73.33%	16.67% (10%)	70%	21.43% (9.52%)	83.33%	27.78% (14.81%)	75%	20.59% (11.76%)	73.08%	18.92% (10.81%)	80%

Figure S11. High-quality of target-specific DMS-MaPseq data

A, Total mismatch percentage on each nucleotide in untreated (DMS-) and DMS-treated (DMS+) 18S rRNA region (179-374nt). The values of one biological replicate for DMS- samples and two biological replicates for DMS+ samples are shown. Data present as means ± standard deviation (SD). B, Ratiometric DMS reactivity (Mismatch/Total) of each A and C were plotted along the 18S rRNA sequence. C,

Phylogenetic structure of *Arabidopsis* 18S rRNA. Nucleotides 179-374 of the phylogenetic structure are color-coded according to the normalized DMS reactivity from the DMS-MaPseq data for one WT shoot FP DMS+ biological replicate as an example. D, High correlation between DMS reactivity and 18S rRNA phylogenetic structure. The DMS reactivity per position normalized to the highest mismatch/total in the tested region, which was set arbitrarily to 1.0. In tested rRNA region (179-374nt), approximate 83-78% (ture positive) of As and Cs that exhibited high DMS reactivity (defined as normalized DMS reactivity ≥ 0.1) in our DMS-MaPseq data corresponded to single-stranded regions in the phylogenetic structure, whereas approximate 84-69% (true negative) of As and Cs that exhibited low DMS reactivity (defined as normalized DMS activity ≤ 0.05) in our DMS-MaPseq data corresponding to based-paired regions in the phylogenetic structure. Of the ~16-27% (false positive) nucleotides (defined as normalized DMS reactivity ≥ 0.1) that were located at base-paired in phylogenetic structure, ~32-55% nucleotides were positioned either at the end of a helix proximal to a bulge or loop, which were known be flexible. Corrected for these positions, the values in parentheses showed higher true positive and lower false positive percentages.

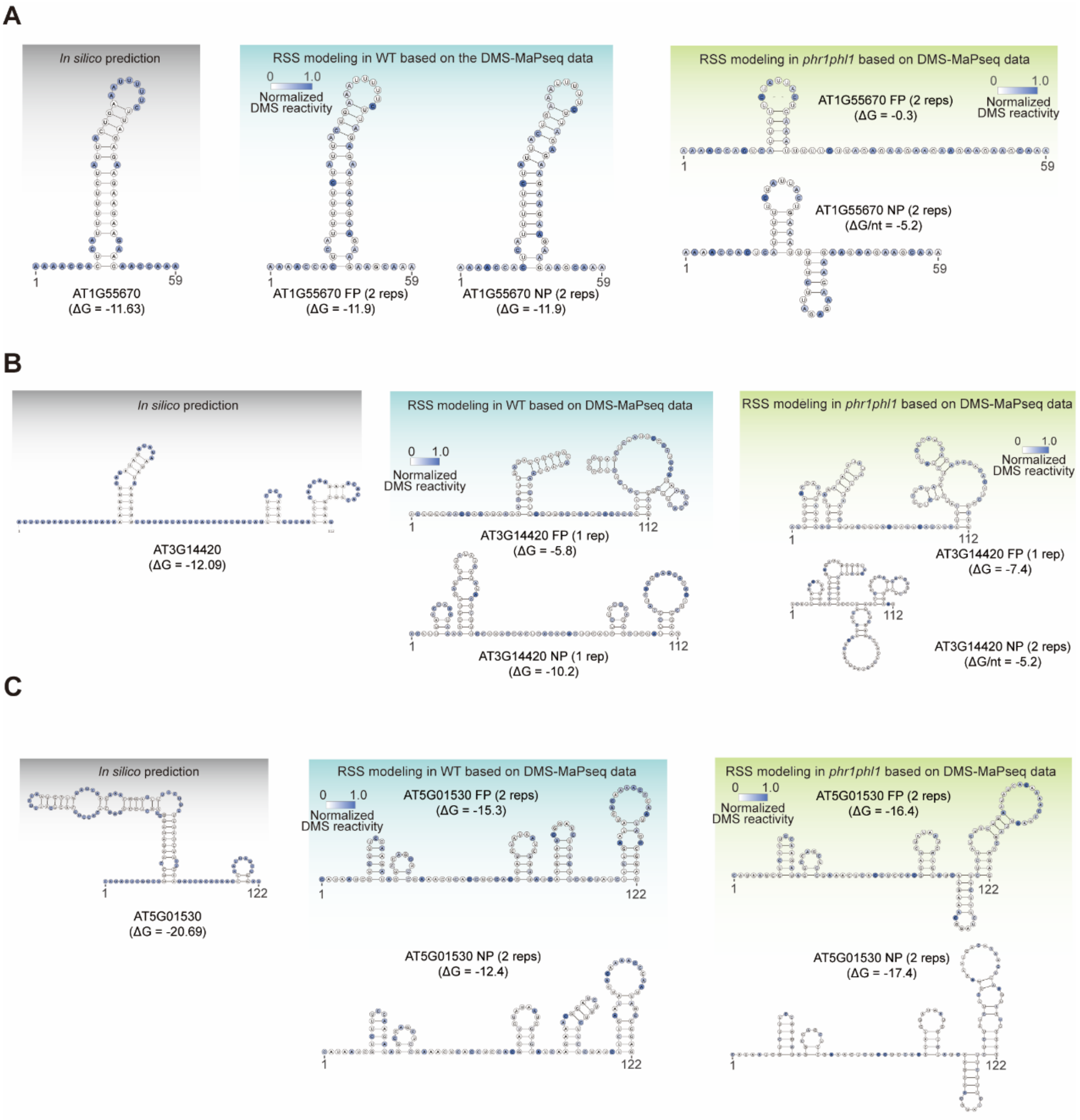

Figure S12. Modeling the RNA secondary structures of the 5'UTRs of PHR-mediated PSR-specific mobile mRNAs based on target-specific DMS-MaPSeq data.

A-C, RSS modeling of the 5'UTR of three PHR-mediated PSR-specific mobile mRNAs based on the target-specific DMS-MaPseq data by RNAstructure or *in silico* prediction by RNAfold in the WT and *phr1 phl1* under FP or NP conditions. For *in silico* prediction, the colored dots indicate the single-stranded structures. For RSS modeling based on DMS-MaPseq data, the shades of color represent the value of normalized DMS reactivity.

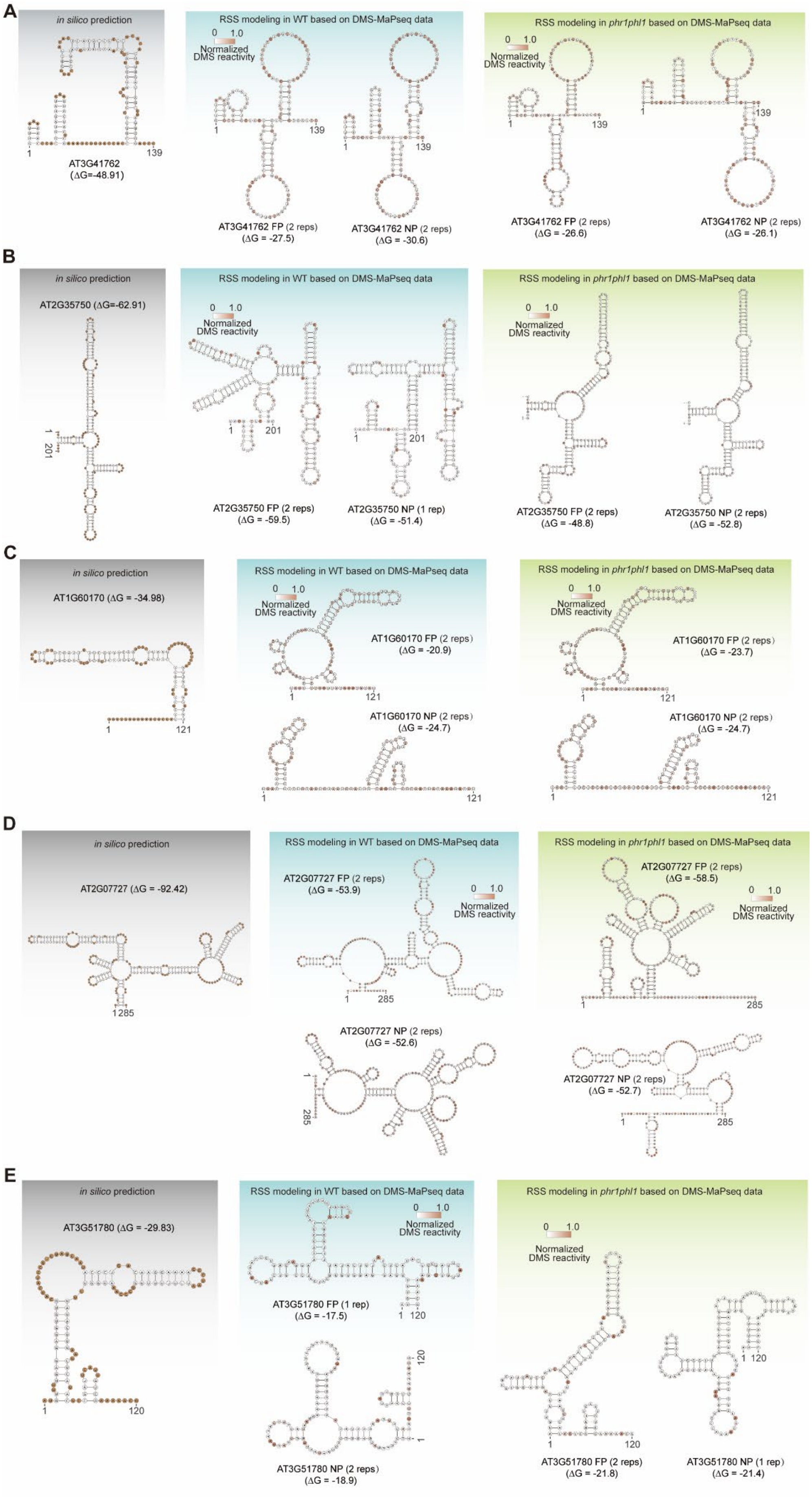

A
in silico prediction
AT3G41762
(ΔG=-48.91)
RSS modeling in WT based on DMS-MaPseq data
0 1.0
Normalized
DMS reactivity
AT3G41762 FP (2 reps)
(ΔG = -27.5)
AT3G41762 NP (2 reps)
(ΔG = -30.6)
RSS modeling in phr1phl1 based on DMS-MaPseq data
AT3G41762 FP (2 reps)
(ΔG = -26.6)
AT3G41762 NP (2 reps)
(ΔG = -26.1)
B
in silico prediction
AT2G35750 (ΔG=-62.91)
RSS modeling in WT based on DMS-MaPseq data
AT2G35750 FP (2 reps)
(ΔG = -59.5)
AT2G35750 NP (1 rep)
(ΔG = -51.4)
RSS modeling in phr1phl1 based on DMS-MaPseq data
AT2G35750 FP (2 reps)
(ΔG = -48.8)
AT2G35750 NP (2 reps)
(ΔG = -52.8)
C
in silico prediction
AT1G60170 (ΔG = -34.98)
RSS modeling in WT based on DMS-MaPseq data
AT1G60170 FP (2 reps)
(ΔG = -20.9)
AT1G60170 NP (2 reps)
(ΔG = -24.7)
RSS modeling in phr1phl1 based on DMS-MaPseq data
AT1G60170 FP (2 reps)
(ΔG = -23.7)
AT1G60170 NP (2 reps)
(ΔG = -24.7)
D
in silico prediction
AT2G07727 (ΔG = -92.42)
RSS modeling in WT based on DMS-MaPseq data
AT2G07727 FP (2 reps)
(ΔG = -53.9)
AT2G07727 NP (2 reps)
(ΔG = -52.6)
RSS modeling in phr1phl1 based on DMS-MaPseq data
AT2G07727 FP (2 reps)
(ΔG = -58.5)
AT2G07727 NP (2 reps)
(ΔG = -52.7)
E
in silico prediction
AT3G51780 (ΔG = -29.83)
RSS modeling in WT based on DMS-MaPseq data
AT3G51780 FP (1 rep)
(ΔG = -17.5)
AT3G51780 NP (2 reps)
(ΔG = -18.9)
RSS modeling in phr1phl1 based on DMS-MaPseq data
AT3G51780 FP (2 reps)
(ΔG = -21.8)
AT3G51780 NP (1 rep)
(ΔG = -21.4)

Figure S13. Modeling the RNA secondary structures of the 5'UTR of non-PHR-mediated mobile mRNAs based on target-specific DMS-MaPSeq data.

A-E, Modeling the RSS of the 5'UTRs of five non-PHR-mediated mobile mRNAs based on the target-specific DMS-MaPseq data by RNAstructure or *in silico* prediction by RNAfold in the WT and *phr1 phl1* under FP or NP conditions. For *in silico* prediction, the colored dots indicate the single-stranded structures. For RSS modeling based on DMS-MaPseq data, the shades of color represent the value of normalized DMS reactivity.

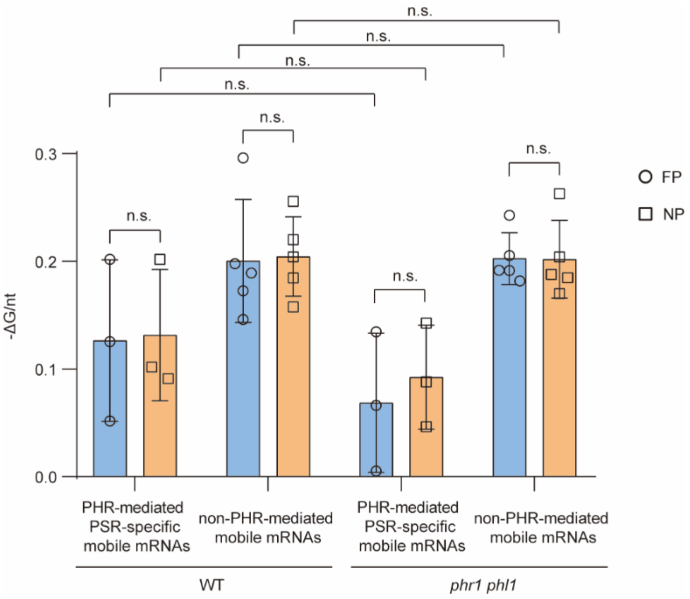

Figure S14. No significant alteration in 5'UTR -ΔG/nt of either PHR-mediated PSR-specific or non-PHR-mediated mobile mRNAs between WT and *phr1 phl1*. Bar plots showing that the -ΔG/nt values of the 5'UTR RSS of either PHR-mediated PSR-specific mobile mRNAs or non-PHR-mediated mobile mRNAs were not significantly altered not only between FP and NP conditions but also between WT and *phr1 phl1* mutants. n.s. no significant. The values are means ± standard deviation (SD), two-tailed Student's *t*-test.

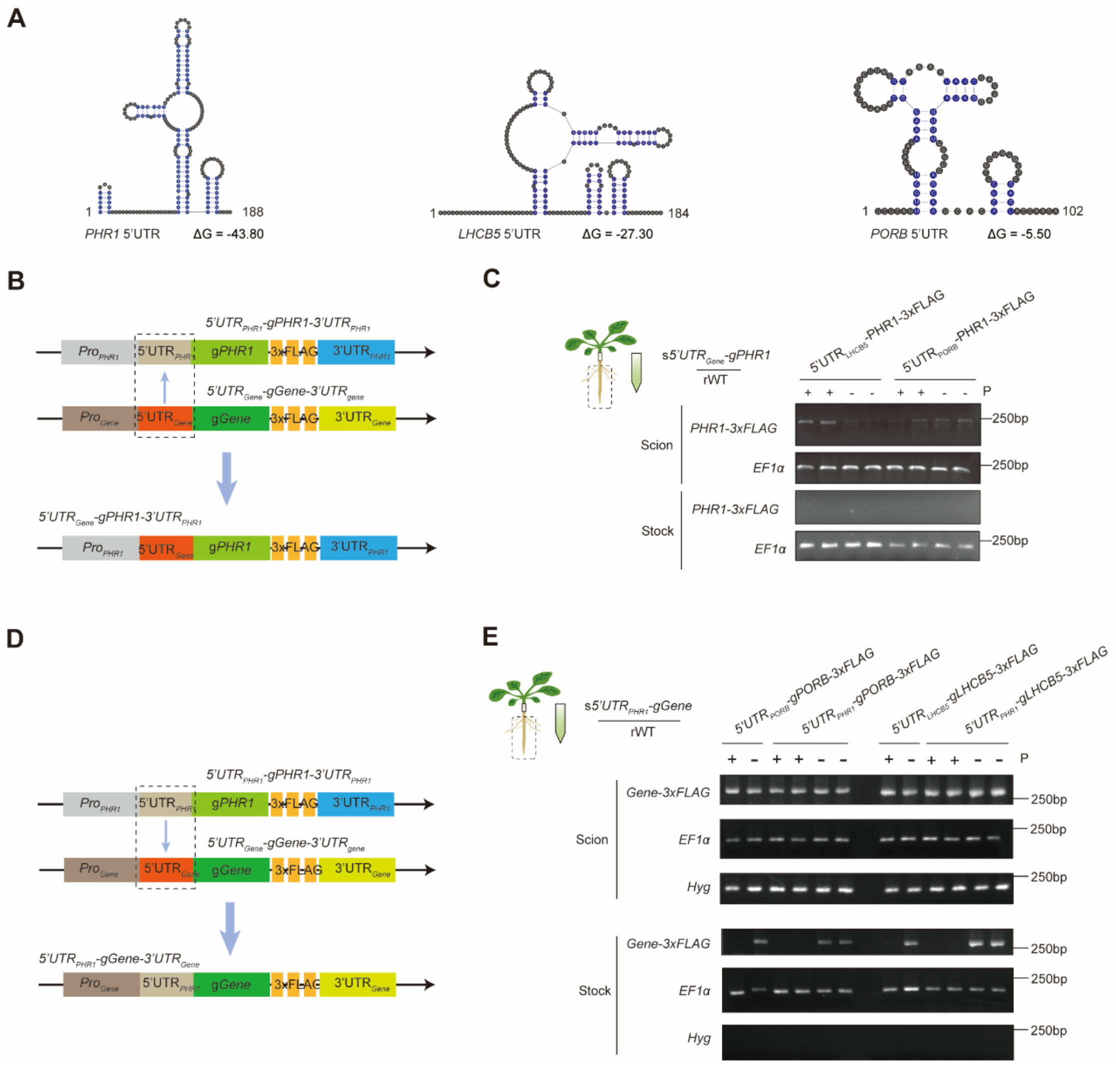

Figure S15. The less-structured 5′ UTR alone is not essential for the mobility of PSR-mediated mobile RNAs.

A, RSS modeling of the 5′ UTRs from the nonmobile RNA *PHR1* and the PHR1-mediated PSR-specific mobile mRNAs *LHCB5* and *PORB* by *in silico* prediction. B and D, Diagram representing the constructs for 5’UTR swapping between *PHR1* and mobile mRNAs. C, RT-PCR analysis showing the non-mobility of the *PHR1-3xFLAG* transcripts under +P and −P conditions, regardless of the 5′ UTR carried by the construct. E, RT-PCR analysis showing the -P-induced long-distance transport from shoot to root of the mobile *Gene-3xFLAG* transcripts were not altered, regardless of the own or *PHR1* 5′ UTR carried by the construct.
